# Assessing European Wheat Sensitivities to *Parastagonospora nodorum* Necrotrophic Effectors and Fine-Mapping the *Snn3-B1* Locus Conferring Sensitivity to the Effector SnTox3

**DOI:** 10.3389/fpls.2018.00881

**Published:** 2018-07-04

**Authors:** Rowena C. Downie, Laura Bouvet, Eiko Furuki, Nick Gosman, Keith A. Gardner, Ian J. Mackay, Camila Campos Mantello, Greg Mellers, Huyen T. T. Phan, Gemma A. Rose, Kar-Chun Tan, Richard P. Oliver, James Cockram

**Affiliations:** ^1^Genetics and Breeding Department, National Institute of Agricultural Botany, Cambridge, United Kingdom; ^2^Plant Sciences Department, University of Cambridge, Cambridge, United Kingdom; ^3^Centre for Crop and Disease Management, Curtin University, Perth, WA, Australia

**Keywords:** *Parastagonospora nodorum*, SnTox3, fungal effector proteins, multi-parent advanced generation inter-cross, genome-wide association scans, high-density SNP genotyping, genetic markers

## Abstract

*Parastagonospora nodorum* is a necrotrophic fungal pathogen of wheat (*Triticum aestivum* L.), one of the world’s most important crops. *P. nodorum* mediates host cell death using proteinaceous necrotrophic effectors, presumably liberating nutrients that allow the infection process to continue. The identification of pathogen effectors has allowed host genetic resistance mechanisms to be separated into their constituent parts. In *P. nodorum*, three proteinaceous effectors have been cloned: *SnToxA*, *SnTox1*, and *SnTox3*. Here, we survey sensitivity to all three effectors in a panel of 480 European wheat varieties, and fine-map the wheat SnTox3 sensitivity locus *Snn3-B1* using genome-wide association scans (GWAS) and an eight-founder wheat multi-parent advanced generation inter-cross (MAGIC) population. Using a Bonferroni corrected *P* ≤ 0.05 significance threshold, GWAS identified 10 significant markers defining a single locus, *Snn3-B1*, located on the short arm of chromosome 5B explaining 32% of the phenotypic variation [peak single nucleotide polymorphisms (SNPs), Excalibur_c47452_183 and GENE-3324_338, -log_10_*P* = 20.44]. Single marker analysis of SnTox3 sensitivity in the MAGIC population located *Snn3-B1* via five significant SNPs, defining a 6.2-kb region that included the two peak SNPs identified in the association mapping panel. Accordingly, SNP Excalibur_c47452_183 was converted to the KASP genotyping system, and validated by screening a subset of 95 wheat varieties, providing a valuable resource for marker assisted breeding and for further genetic investigation. In addition, composite interval mapping in the MAGIC population identified six minor SnTox3 sensitivity quantitative trait loci, on chromosomes 2A (*QTox3.niab-2A.1, P-*value = 9.17^-7^), 2B (*QTox3.niab-2B.1*, *P* = 0.018), 3B (*QTox3.niab-3B.1*, *P* = 48.51^-4^), 4D (*QTox3.niab-4D.1*, *P* = 0.028), 6A (*QTox3.niab-6A.1, P* = 8.51^-4^), and 7B (*QTox3.niab-7B.1*, *P* = 0.020), each accounting for between 3.1 and 6.0 % of the phenotypic variance. Collectively, the outcomes of this study provides breeders with knowledge and resources regarding the sensitivity of European wheat germplasm to *P. nodorum* effectors, as well as simple diagnostic markers for determining allelic state at *Snn3-B1*.

## Introduction

The necrotrophic pathogen *Parastagonospora* (synonyms: *Septoria*, *Stagonospora*, *Phaeosphaeria*) *nodorum* (Berk.) Quaedvlieg, Verkley, and Crous is the causal agent of the disease SNB and glume blotch in wheat (*Triticum aestivum* L.), a disease of significant economic importance in Australia, Europe, North America, and Northern Africa (Friesen et al., 2005; Oliver et al., 2012; Quaedvlieg et al., 2013). The visual symptoms of SNB are chlorosis and necrosis of the leaf tissue, as well as discoloration of the glumes, often in the form of lesions (Solomon et al., 2006). These symptoms reduce the leaf surface area capable of photosynthesis, limiting overall crop growth, with SNB shown to result in grain yield losses of up to 31% (Bhathal and Loughman, 2003). *P. nodorum* is thought to derive nutrients from dead plant tissue, utilizing fungal effector proteins, previously known as host-specific (or selective) toxins, to induce a hypersensitive response in the host, which takes the form of programmed cell death (Friesen et al., 2007; Liu et al., 2009; Oliver et al., 2012). The necrotic response in the sensitive host is hypothesized to facilitate pathogen colonization, promoting infection and ultimately providing a rich nutrient source, via cell death (Oliver and Solomon, 2010; Vincent et al., 2012). This is known as effector-triggered susceptibility and is genetically induced via an “inverse gene for gene system” (Friesen et al., 2007). The identification of effector proteins has led to a paradigm shift in the approach to tackle these types of pathogens, as the host–pathogen interactions can be broken down into their constituent parts. Consequently, targeted breeding could then be used to eliminate host sensitivity on an effector by effector basis.

Effector proteins were described for the first time with regards to a host–pathogen interaction between *Alternaria alternata* (a necrotroph) and *Pirus serotine* (Tanaka, 1933). However, the first protein effector described in a necrotrophic pathogen was PtrToxA from the wheat tan spot pathogen, *Pyrenophora tritici-repentis*, which triggers necrosis in wheat lines carrying susceptible alleles at the *Tsn1* locus (Ballance et al., 1989; Tomas et al., 1990; Faris et al., 1996). A near identical effector, SnToxA, was discovered in *P. nodorum*, with the corresponding host sensitivity locus also being *Tsn1* (Liu et al., 2006). *Tsn1*, located on the long arm of chromosome 5B, has been cloned and encodes an intracellular protein with a serine/threonine protein kinase (S/TPK) domain, a nucleotide-binding site (NBS), and leucine-rich repeats (LRRs), with deletion of *Tsn1* resulting in SnToxA insensitivity (Faris et al., 2010). Similarly, the purification and subsequent isolation of the *P. nodorum* effector, SnTox1, allowed identification of the corresponding wheat sensitivity locus, *Snn1*, on the short arm of chromosome 1B (Liu et al., 2004, 2012; Cockram et al., 2015). Map-based cloning found *Snn1* to encode a wall-associated kinase (WAK), with yeast two-hybrid analysis showing the Snn1 and SnTox1 proteins interact directly *in vitro* (Shi et al., 2016b), unlike *Tsn1* and SnToxA (Faris et al., 2010). Given the nature of their corresponding wheat sensitivity loci, it is hypothesized that SnToxA and SnTox1 activate the wheat pathogen-associated molecular pattern (PAMP)-triggered immunity (PTI) and effector-triggered immunity (ETI) pathways, which for biotrophic pathogens protect against pathogen infection. However, as *P. nodorum* is a necrotrophic pathogen, the triggering of these pathways, which induce necrosis and cell death, promotes *P. nodorum* growth and propagation (Shi et al., 2016b). Wheat varieties carrying both *Tsn1* and *Snn1* show higher levels of necrosis than those varieties carrying either *Tsn1* or *Snn1* alone (Chu et al., 2010), indicating that the hijacking of both the PTI and ETI pathways for necrotrophic effector triggered susceptibility supports pathogen survival and reproduction (Shi et al., 2016b).

Characterization of a third *P. nodorum* effector, SnTox3, led to the identification of its corresponding wheat sensitivity locus, *Snn3* (more recently termed *Snn3-B1*), located on the short arm of chromosome 5B. Culture filtrate containing SnTox3 was produced using a wildtype pathogen isolate, SN15, and host sensitivity was mapped using the BR34 × Grandin wheat population, accounting for 17% of the phenotypic variation (Friesen et al., 2008). This agrees with data from the doubled haploid mapping population, Calingiri × Wyalkatchem, which identified the SnTox3 sensitivity locus *QSnb.fcu-5BS*, as well as a minor SnTox3 sensitivity QTL on the long arm of chromosome 4B, *Qsnb.cur-4BL* (Phan et al., 2016).

Understanding the effector sensitivities of wheat varieties, and the genetic determinants controlling wheat sensitivity, allows informed manipulation of alleles and germplasm within wheat breeding programs. Here, we survey a panel of 480 predominantly British winter wheat varieties for sensitivity to SnToxA, SnTox1, and SnTox3, and use this AM panel in concert with a MAGIC population to fine-map *Snn3-B1*, and to identify additional minor QTL for SnTox3 sensitivity.

## Materials and Methods

### Wheat Germplasm and High-Density Genotyping

Two bread wheat (*T. aestivum* L.) populations were used for effector sensitivity screening and genetic mapping. The first was an AM panel, representing a diverse collection of 480 elite, predominantly British, wheat varieties drawn from historic collections and National Lists, encompassing varieties released between 1916 and 2007 (Supplementary Table S1). Of these, 420 were released or marketed within the United Kingdom; however, many of these were bred for initial release outside of the United Kingdom (data not available). The remaining 60 varieties do not have a United Kingdom Application For Protection (AFP) number, and so where country of origin information was not available, were assumed to either be non-UK, or represent accessions that predate the application process (represented in Supplementary Table S1 as accessions beginning with the prefix “U”). The majority of the AM panel represent British varieties (330 lines, 68% of the total collection), followed by 51 French (10%), 37 German (8%), and 19 Dutch varieties. The remaining 17 varieties with country information come from Belgium, Canada, Denmark, Sweden, Switzerland, and United States. For 26 varieties, country of origin was not known. The population was previously genotyped using the Illumina iSelect 90,000 feature wheat SNP array (Wang et al., 2014), resulting in 26,018 polymorphic SNPs with a minor allele frequency ≥ 3% (available via http://www.niab.com/pages/id/326/Resources). The second was an eight-founder MAGIC population, termed the “NIAB Elite MAGIC” population (Mackay et al., 2014), the founders of which (cvs. Alchemy, Brompton, Claire, Hereward, Rialto, Robigus, Soissons, and Xi19) were selected for their high seed yield, disease resistance, and their range of end-use qualities. The founders were intercrossed in a simple replicated funnel crossing scheme over three generations, with individuals from the eight-way families subsequently selfed over four generations through single seed descent to produce >1,000 recombinant inbred lines. A subset of these F_5_ recombinant inbred lines were genotyped using the 90,000 feature SNP array detailed above, resulting in 20,643 polymorphic markers (Gardner et al., 2016). These data allowed the development of a high-resolution genetic map consisting of 18,601 markers mapped using 643 MAGIC lines (Gardner et al., 2016). The remaining 2,042 SNPs were not mappable, due largely to segregation distortion and/or dominance (Gardner et al., 2016).

### Effector Protein Production, Wheat Phenotyping, and Pedigree Analysis

SnTox1 and SnTox3 were expressed in *Pichia pastoris*, as previously described (Tan et al., 2014). For SnToxA, heterologous expression was conducted in *Escherichia coli* BL21E using the pET21a expression vector as described in Tan et al. (2012). Protein preparations were desalted in 20 mM pH 7.0 sodium phosphate, freeze-dried for storage, and subsequently re-suspended in ultra-pure water and stored at 4°C prior to use. The AM and MAGIC lines were grown in 96-well trays with fine/medium compost (M3) in a heated and lit glasshouse at 20°C/17°C day/night with a 16-h photoperiod. Each line was represented by three to four replicates, and each MAGIC founder by eight replicates, with experimental design carried out using MATLAB (MATLAB, The MathWorks Inc., Natick, Massachusetts, United States) or R/blocksdesign^1^. For the AM panel, randomization was performed using a custom software routine written in the MATLAB programming environment, to include three biological replicates of each line. For the MAGIC population, the experimental design was split into four blocks, each block containing one replicate of each line and two replicates of each of the parents, with line positions randomized within each block. Therefore, a total of four biological replicates of each line and eight biological replicates of each parent were included. Infiltration on the AM and MAGIC populations was undertaken on seedlings at Zadoks growth stage (GS) 12 (Zadoks et al., 1974), as previously described (Tan et al., 2012). Briefly, a 1-ml plastic syringe was used to infiltrate approximately 50 μl of either SnToxA, SnTox1, or SnTox3 suspension into the first leaf, with the extent of the leaf infiltration region marked with a non-toxic pen. Seven days following infiltration, the plants were visually evaluated for SnTox3 effector sensitivity on a scale of 0 (insensitivity, no symptoms) to 4 (extensive necrosis; Tan et al., 2012). A water control was also used to establish a symptom baseline to evaluate possible damage due to the infiltration process. Mean sensitivity scores were calculated per line for subsequent analysis. Wheat pedigree information was obtained from public sources, and displayed using the software Helium v.1.17.08.14 (Shaw et al., 2014).

### Statistical Analyses and Bioinformatics

Effector phenotypic data were analyzed using R (R Core Team, 2013) to determine mean sensitivity scores for each variety and variance within variety. For each toxin, the heritability of line means (broad sense) was calculated by first estimating components of variation from ANOVA (in the AM panel) or REML (in MAGIC) while taking into account all features of the experimental designs. Heritability was then estimated as *h*^2^ = σ^2^*_G_*/(σ^2^*_G_* + σ^2^e) where σ^2^*_G_* is the genetic variation between line means and σ^2^e is the error variance appropriate to those means. Calculations were carried out in GenStat (VSN International, 2011) and the package lme4 (Bates et al., 2015) in R. GWAS using the AM panel was undertaken using the efficient mixed model association (EMMA) algorithm (Kang et al., 2008) using a compressed mixed linear model (CMLM; Zhang et al., 2010) which includes both fixed and random effects, implemented with the Genome Association and Prediction Integrated Tool (GAPIT) package (Lipka et al., 2012) in R. The genotyping and quality control of the 26,016 SNPs, and the generation of the kinship matrix, is previously described, with the majority of the population structure evident due to spring or winter seasonal growth habit (Gardner et al., submitted). For GWAS, Bonferroni corrected *P* = 0.05 and *P* = 0.01 significance thresholds were used, termed here “significant” and “highly significant,” respectively. For MAGIC, correction for multiple testing was carried out using R/qvalue using corrected threshold of 0.05. MAGIC genetic analyses were undertaken using two approaches.

(1) SMA: a simple linear model test in R/lme4 using all 20,643 SNPs. After finding of a major QTL, the analysis was repeated with the major QTL as a covariate.
(1) Haplotype analysis using a subset of 7,369 uniquely mapped SNPs from the MAGIC genetic map (Gardner et al., 2016).

Founder haplotype probabilities were computed with the “mpprob” function in R/mpMap (Huang and George, 2011), implemented in R/qtl (Broman et al., 2003), using a threshold of 0.5. QTL analysis with haplotypes was carried out (a) by linear mixed model using all mapped markers and (b) by CIM using the mpIM function in R/mpMap, with either 0, 5, or 10 covariates. The function sim.sigthr in R/mpMap was used to conduct 100 simulations using the dataset to obtain an empirical QTL significance threshold at a *P* threshold of 0.05. A full QTL model was then fitted with all QTL using R/fit.mpQTL. For the AM panel, the difference between homozygous marker classes was estimated as twice the linear regression coefficient from a regression of the trait on the marker classes (coded 0, 1, and 2 with 1 being the heterozygous genotype). The coefficient of determination (or *r*-squared) was used as a measure of the proportion of variance explained by the marker. Genetic markers were anchored using BLASTn (Altschul et al., 1990) against the wheat cv. Chinese Spring 42 IWGSC RefSeq v1.0 physical map (pre-publication data made available under the IWGSC General Data Access Agreement via https://wheat-urgi.versailles.inra.fr/), and where explicitly stated in the text, against the TGACv1 cv. Chinese Spring 42 physical map (Clavijo et al., 2017). In the case of hits of equal match on multiple homoeologs, chromosome allocation followed that assigned by the genetic map (Gardner et al., 2016), where possible. Nomenclature for the QTL discovered in this study follows that recommended by the Catalog of Gene Symbols for Wheat (McIntosh et al., 2008). Protein domains were identified using Pfam 31.0 (Finn et al., 2016).

### KASP Marker Development

Single nucleotide polymorphisms were converted to the Kompetitive Allele-Specific PCR (KASP) genotyping platform (LGC Genomics, United Kingdom). SNP flanking DNA sequences were used to design KASP primers using the software PolyMarker (Ramirez-Gonzalez et al., 2015). Genomic DNA was extracted from seedling leaves harvested from a subset of the AM panel using a modified Tanksley protocol (Fulton et al., 1995), DNA concentrations determined using a Nanodrop 200 spectrophotometer (Thermo Scientific), and diluted to a final concentration of 10 ng/μl using sterile PCR-grade water. KASP genotyping was undertaken by a service provider following the manufacturer’s guidelines (LGC Genomics), returned as .csv files, analyzed with SNP Viewer v.1.99^2^, and compared against the corresponding SNP calls from the Illumina 90k SNP array.

## Results

### *P. nodorum* Effector Sensitivity Phenotyping

**FIGURE 1:**
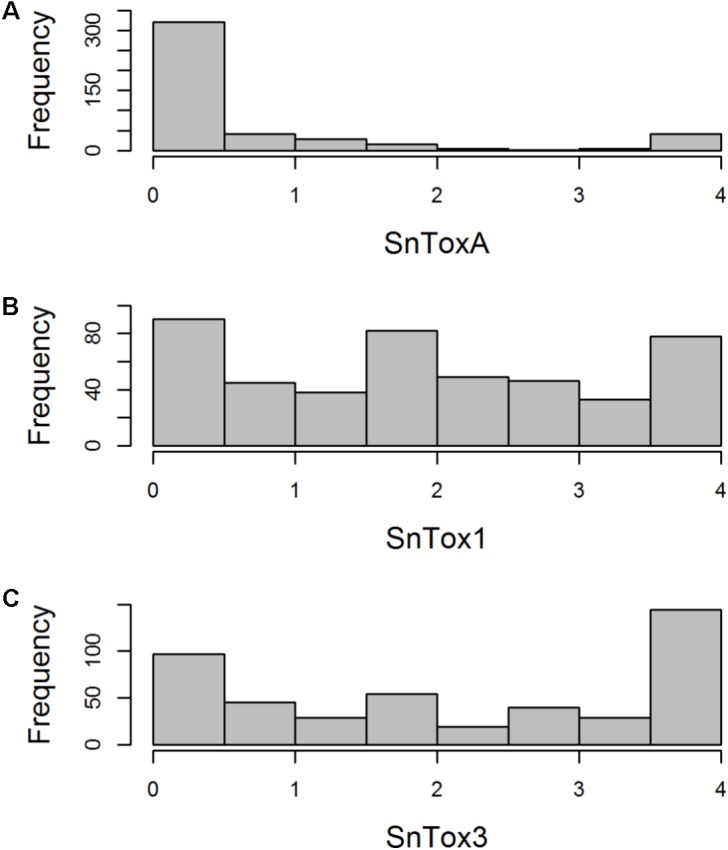
Histogram of host sensitivity to *P. nodorum* effectors **(A)** SnToxA, **(B)** SnTox1, and **(C)** SnTox3 in the AM panel of 480 northwest European wheat varieties. Sensitivity score (0 = insensitive, 4 = sensitive) for each accession represents a mean of four biological replicates.

**FIGURE 2:**
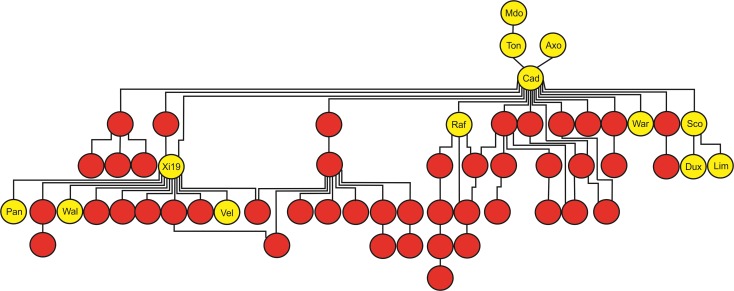
Tracking the transmission of SnToxA sensitivity in the wheat pedigree around the Axona × Tonic pedigree. SnToxA sensitive (score ≥ 3) = yellow. SnToxA insensitive (score < 1) = red. Variety names listed only for SnToxA sensitive varieties in the pedigree: Mdo = Maris Dove, Ton = Tonic, Axo = Axona, Cad = Cadenza, Raf = Raffles, War = Warlock_24, Sco = Scorpion_25, Dux = Duxford, Lim = Limerick, Pan = Panorama, Wal = Walpole, Vel = Velocity, and Cur = KWS Curlew.

The AM panel, consisting of 480 varieties and breeding lines released between 1916 and 2007, was phenotyped for sensitivity to SnToxA, SnTox1, and SnTox3 via leaf infiltration, and the severity of host response scored using a 0 (insensitive) to 4 (extensive necrosis) scale (**Figure 1** and Supplementary Table S1). Broad sense heritability for effector sensitivity was found to be highest for SnTox3 (*h*^2^ = 0.92), followed by SnToxA (*h*^2^ = 0.83) and SnTox1 (*h*^2^ = 0.77). For SnToxA, a separation into insensitive/weakly sensitive (0 ≤ score < 1) and strongly sensitive (score ≥ 3) was observed, accounting for 73% (338/460 varieties) and 10% (47/460 varieties) of all lines successfully screened, respectively (**Figure 1A**). While spring cultivars represent just 6% of the complete AM panel, 23% (11/47 cultivars) of all SnToxA sensitive lines are spring cultivars, as is the oldest SnToxA sensitive variety, Garnet (released in 1926). Analysis of the wheat pedigree (**Figure 2**) shows the SnToxA sensitive varieties Axona (released in 1984) and Tonic (released in 1983) to be the most prominent in the transmission of strong sensitivity (due to the prominence of their offspring Cadenza in the wheat pedigree), accounting for 23% of varieties with a sensitivity score ≥ 3. Indeed, the four most recent SnToxA sensitive varieties (Duxford, KWS Curlew, Limerick, and Velocity, released in 2006) all have Cadenza in their pedigree. Response to SnTox1 is more evenly spread across sensitivity classes, with 24% insensitive (111/461 varieties), 28% sensitive (130/461), and 48% (220/461) showing intermediate sensitivity (1 ≥ score < 3; **Figure 1B**). No pedigree relationships of note were observed for SnTox1 and SnTox3 sensitivity. The most common effector sensitivity identified in the AM population was to SnTox3, with 42% of varieties found to be strongly sensitive (192/457), including the recent varieties Cocoon, KWS Santiago, Orator, Rainbow, and Tuxedo, all released in 2007. Insensitivity to SnTox3 was found in 25% (114/457) of lines, including six released in 2007, while intermediate sensitivity was observed in 33% of varieties. All possible combinations of sensitivity between the three effectors were identified, with 18 varieties displaying high sensitivity (score ≥ 3) to all three effectors.

### Genetic Analysis of SnTox3 Sensitivity Using the AM Panel

Initially, the precision of the AM panel was assessed empirically by undertaking GWAS for SnToxA sensitivity, known to be due to allelic variation at the gene underlying *Tsn1.* Using a data matrix of 26,018 SNPs across 480 varieties, and a Bonferroni corrected *P* = 0.01 significance threshold (-log_10_*P* = 6.41), GWAS identified 30 highly significant markers associated with SnToxA sensitivity (Supplementary Table S2A). These accounted for between 25% and 60% of the total variation (average 38%) with differences in score between the two homozygous classes ranging from 1.5 to 3.1 (mean 2.5). Of the eight most significant markers (-log_10_*P* ≥ 31.34), six are located within a gene model encoding a potassium transporter (TraesCS5B01G368500), just two genes proximal to the *S/TPK-NBS-LRR* gene underlying *Tsn1* (Faris et al., 2010). Similarly, GWAS of SnTox1 sensitivity identified seven highly significant (-log_10_*P* > 6.41) SNPs. These accounted for between 8.3% and 14.3% of the variation (average 11.6%) with differences in score between the two homozygous classes ranging from 0.9 to 1.6 (mean 1.4). All seven SNPs were located at the *Snn1* locus, with SNP Excalibur_c21898_1423 located 25 genes distant from the *WAK* gene underlying *Snn1* in cv. Chinese Spring (TraesCS1B01G004100; Supplementary Table S2B).

**FIGURE 3:**
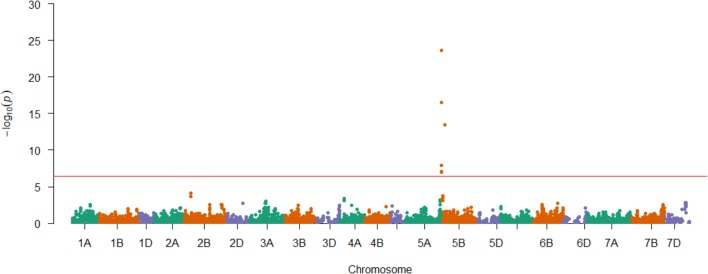
Genetic mapping of SnTox3 sensitivity in the AM panel. SNPs are ordered according to the genetic map (Gardner et al., 2016). Significant SNPs that were unmapped in the genetic map (Gardner et al., 2016) are included on the relevant chromosome (5B) via their physical map positions (IWGSC RefSeq v1.0), relative to those of the genetically mapped chromosome 5B SNPs. The Bonferroni corrected *P* = 0.01 significance threshold is indicated in red (–log_10_*P* = 6.41).

Having demonstrated the utility of the AM panel, we proceeded to use SnTox3 sensitivity phenotypic data to undertake GWAS, identifying 14 significant SNPs (**Table 1A** and **Figure 3**). Of these, seven were located within a single region on the short arm of chromosome 5B in the IWGSC RefSeq v1.0 physical map. While the remaining three SNPs (wsnp_Ex_c9301_15450818, IACX7443, and Ra_c68425_1406) returned hits on unallocated chromosomes in IWGSC RefSeq v1.0, all three were localized to chromosome 5B on the TGACv1 wheat reference sequence (**Table 1A**), and have been previously allocated to the short arm of chromosome 5B by treating the SNP as a trait, and locating its estimated position by trait mapping (Gardner et al., 2016). The most significant SNPs were Excalibur_c47452_183 and GENE-3324_338 (-log_10_*P* = 20.44) and explained 32% of the phenotypic variation, explaining a phenotypic difference between homozygous allele classes of 2.1. Anchoring previous markers identified as flanking *Snn3-B1* to the IWGSC RefSeq v1.0 wheat genome reference sequence confirmed we had identified *Snn3-B1* in the GWAS panel. Using our peak *Snn3-B1* marker Excalibur_c47452_183 as a cofactor in GWAS found no additional significant genetic loci outside of the *Snn3-B1* region (Supplementary Table S3).

**Table 1:** Significant SNPs (*P* ≤ 0.05, Bonferroni corrected) for SnTox3 sensitivity identified in **(A)** the AM panel and **(B)** single marker analysis (SMA) on 20,643 SNPs in the MAGIC population, listing the SNPs with effect > 1.0 (see Supplementary Table S4 for details of all 114 significant SNPs).

SNP	MAF	-log_10_*P*	Chr, SNP position (bp)	IWGSC RefSeq v1.0 gene model	Gene annotation
**A**
Excalibur_c47452_183	0.194	23.62	5B, 6654166	TraesCS5B01G005100	Ubiquitin conjugating enzyme E2
GENE-3324_338	0.194	23.62	5B, 6647920^†^	TraesCS5B01G005000	Nucleotide triphosphate hydrolase
BobWhite_c4838_58	0.194	23.62	5B, 6654053	TraesCS5B01G005100	Ubiquitin conjugating enzyme
BS00091518_51	0.194	23.62	5B, 6648547	TraesCS5B01G005000	Nucleotide triphosphate hydrolase
BS00091519_51	0.194	23.62	5B, 6648567	TraesCS5B01G005000	Nucleotide triphosphate hydrolase
BS00064297_51b	0.272	16.52	5B, 6974807	TraesCS5B01G005600	Transmembrane protein, putative (DUF594)
BS00064298_51b	0.272	16.52	5B, 6974825	TraesCS5B01G005600	Transmembrane protein, putative (DUF594)
Ex_c1846_1818b	0.449	14.87	5B, 64632597	TraesCS5B01G059000	Transmembrane protein, putative (DUF594)
Ex_c1846_1818a	0.271	13.46	5B, 64736555	TraesCS5B01G059000	Transmembrane protein, putative (DUF594)
RAC875_c7582_680	0.124	7.88	5B, 2058821	TraesCS5B01G002000LC	None
Ku_c10387_272	0.124	7.88	5B, 232228	TraesCS5B01G000600	Microtubule-associated protein 70-2
RAC875_c39204_91	0.157	7.11	5B, 6852650	None	None
BS00064297_51a	0.156	7.02	5B, 6974807	TraesCS5B01G005600	Transmembrane protein, putative (DUF594)
BS00064298_51a	0.156	7.02	5B, 6974825	TraesCS5B01G005600	Transmembrane protein, putative (DUF594)
**B**			
Excalibur_c47452_183		15.7^‡^	5B, 6654166	TraesCS5B01G005100	Ubiquitin conjugating enzyme E2
GENE-3324_338		15.7^‡^	5B, 6647920^†^	TraesCS5B01G005000	Nucleotide triphosphate hydrolase
BobWhite_c4838_58		15.7^‡^	5B, 6654053	TraesCS5B01G005100	Ubiquitin conjugating enzyme E2
BS00091518_51		15.7^‡^	5B, 6648547	TraesCS5B01G005000	Nucleotide triphosphate hydrolase
BS00091519_51		15.7^‡^	5B, 6648567	TraesCS5B01G005000	Nucleotide triphosphate hydrolase


*MAF, minor allele frequency (AM panel only); Chr, chromosome; U, chromosome unknown. ^†^While chromosome 5A homoeolog represented a better BLASTn hit, details for the 5B homoeologs are presented here, as this SNP has previously been shown to be located on chromosome 5B by using the SNP as a trait, and localizing to a chromosome by trait mapping (Gardner et al., 2016). ^‡^*P-*values < 2.2^-16^, so -log_10_*P* set arbitrarily to > 15.7 here.*

### Genetic Analysis of SnTox3 Sensitivity Using MAGIC

While screening the AM panel for SnTox3 sensitivity, the eight founders of the NIAB Elite MAGIC population were found to contrast for SnTox3 sensitivity (Supplementary Table S1). Accordingly, 643 lines of the MAGIC population (3–4 reps/line), as well as the eight founders (8 reps/line), were subsequently phenotyped for SnTox3 sensitivity. The sensitivity scores of the parents ranged from 0 (Alchemy, Claire, Robigus) to 4 (Hereward, Rialto, Soissons, Xi19), with Brompton displaying intermediate sensitivity (1.93). SnTox3 sensitivity scores in the MAGIC progeny ranged from 0 to 4, with the majority of lines displaying high sensitivity (score ≥ 3.5, 42.5% lines) or no sensitivity (score ≤ 0.5, 27.5% lines; Supplementary Figure S1). Heritability for SnTox3 sensitivity in the MAGIC population was calculated to be *h*^2^ = 0.95. Initially, the 643 MAGIC lines along with the 20,643 mapped and unmapped SNPs were used for SMA using a simple linear model test, identifying 114 significant (*P <* 0.05) markers (Supplementary Table S4). Of these, the five most significant SNPs, with predicted allelic effects > 1, are the same as the five most significant markers identified in the AM panel (Excalibur_c47452_183, GENE-3324_338, BobWhite_c4838_58, BS00091518_51, and BS00091519_51; **Table 1B**). SMA identified four additional QTL. The first was on chromosomes 2B (termed here *QTox3.niab-2B.1*, *P* = 0.023), located at 356.66 cM by SNP Kukri_c9898_1766. The second, at 40.61 cM on chromosome 4D (*QTox3.niab-4B.1*, *P* = 0.037), was identified by SNP BS00036421_51, the third (*QTox3.niab-6B.1*) was identified by three SNPs on chromosome 6B, with wsnp_Ku_c2119_4098330 showing the highest significance (*P* = 0.003), and the fourth (*QTox3.niab-7B.1*) was identified by four chromosome 7B SNPs, with BS00022127_51 showing the highest significance (*P* = 0.038). Using the most significant peak SNP GENE_3324_338 as a covariate in SMA analysis did not identify any additional genetic loci, and resulted in the disappearance of all four minor QTL.

Additionally, CIM using 0, 5, and 10 covariates was undertaken, using the 7,369 uniquely mapped SNPs from the MAGIC genetic map (Gardner et al., 2016). As well as identifying *Snn3-B1* on chromosome 5B (*P*-values for all analyses ≤ 2.2^-16^, accounting for ≥16.95% of the phenotypic variation), we again detected *QTox3.niab-2B.1* (*P* = 0.047, 2.3% variation explained, detected with 0 covariates only) and *QTox3.niab-7B.1* (*P* = 0.025, 1.6% variation explained, SNP detected with 0 covariates only). In addition, three further QTL were discovered, distinct to those identified by SMA (**Table 2**). The first, *QTox3.niab-2A.1*, mapped to chromosome 2A at 234.62 cM (SNPs BS00070979_51 and Excalibur_c20478_641, positioned at ∼758 Mb) with a *P-*value = 9.17^-7^, and explained 6.0% of the variation with 0 covariates. The second, *QTox3.niab-3B.1*, was located on 3B at 84.11 cM (*P* = 48.51^-4^, SNPs wsnp_Ex_c11246_18191331, wsnp_Ex_c22401_31592784, ∼68 Mb), and explained 3.1% of the variation (only found with 5 or 10 covariates). Finally, *QTox3.niab-6A.1* at 65.6 cM on chromosome 6A explained 4.2% of the variation with 0 covariates (*P* = 8.51^-4^, SNPs BobWhite_c13839_135 and IACX7801, ∼22 Mb; Supplementary Table S7).

**Table 2:** Significant SnTox3 sensitivity QTL identified by CIM in the MAGIC population, incorporating 0, 5, and 10 covariates in the analysis (labeled below as cov 0, cov 5, and cov 10, respectively).

Cov 0			Cov 5			Cov 10		
		
Chr, cM	*P*-value, % Va	SNPs flanking QTL peak	Chr, cM	*P-*value, % Va	SNPs flanking QTL peak	Chr, cM	*P-*value, % Va	SNPs flanking QTL peak
2A, 234.62	9.17^-7^, 6.0	BS00070979_51, Excalibur_c20478_641	2A, 235.12	1.18^-5,^ 4.9	Excalibur_c20478_641, Tdurum_contig56321_232	2A, 234.62	3.22^-6^, 2.8	BS00070979_51, Excalibur_c20478_641
2B, 379.61	4.7^-3^, 2.3	BS00064483_51, Kukri_c1526_666						
			3B, 84.11	8.51^-4^, 3.1	wsnp_Ex_c11246_18191331, wsnp_Ex_c22401_31592784	3B, 84.11	7.96^-4^, 3.1	wsnp_Ex_c11246_18191331, wsnp_Ex_c22401_31592784
5B, 1.27	3.33^-16,^ 15.5	BS00015136_51, GENE-3277_145	5B, 3.29	0, 17.0	BS00025784_51, BS00065732_51	5B, 3.29	0^∗^, 17.0	BS00025784_51, BS00065732_51
6A, 65.6	6.16^-5^, 4.2	BobWhite_c13839_135, IACX7801				6A, 65.6	3.53^-4^, 3.2	BobWhite_c13839_135, IACX7801
7B, 9.43	0.025, 1.6	BS00022127_51, Kukri_c67849_109						


*% Va, percent variation explained. QTL identified by two or more covariate analysis methods are reported in the manuscript, using *P-*values and SNP information from the analysis with the least number of covariates. Chromosome (Chr) and cM positions are from the NIAB Elite MAGIC genetic map (Gardner et al., 2016). ^∗^<2.2–16.*

### Development of KASP Genetic Markers for *Snn3-B1*

**FIGURE 4:**
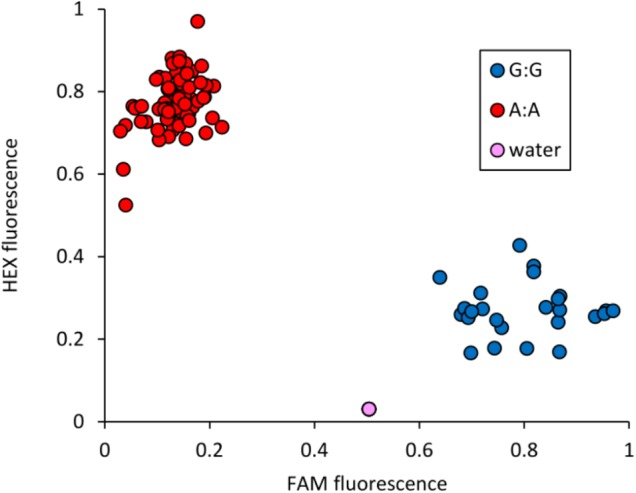
Validation of KASP marker Excalibur_c47452_183 (SNP assayed = A/G), closely linked to *Snn3-B1.* Allele-specific primer A: 5′-GAAGGTCGGAGTCAACGGATTaaggctggctggcgagtA-3′. Allele-specific primer B: 5′-GAAGGTGACCAAGTTCATGCTaaggctggctggcgagtG-3′. Common primer: 5′-tgagggggcatcgaatcG-3′. Tails for ligation of fluorophores are included in uppercase at the 5′ end of the allele-specific primers.

The peak SNP identified in both the AM panel by GWAS and MAGIC population by SMA was Excalibur_c47452_183. This marker was selected for conversion from the 90k SNP array to the KASP genotyping platform, a single-plex technology that allows flexible, low-cost use for marker-assisted breeding and research. Primers were designed and tested on a subset of 95 varieties from the AM panel (**Figure 4** and Supplementary Table S5). Comparison of Excalibur_c47452_183 allele calls from KASP genotyping with those returned by the 90k array genotyping of the AM panel found perfect correspondence between the two, indicating robust conversion to the KASP platform (Supplementary Table S5). This SNP provides good, but not perfect, prediction of SnTox3 sensitivity phenotype in the AM panel (**Table 3** and Supplementary Table S1).

**Table 3:** Allele call for SNP marker Excalibur_c47452_183 versus SnTox3 sensitivity score in the AM panel.

	SnTox3	SnTox3	Total no. of
	sensitivity < 4	sensitivity = 4	varieties^†^
Allele A:A	317	43	360
Allele G:G	6	91	97
Total no. of varieties^†^	323	134	457


*^†^Accessions for which phenotypic data were available.*

### Analysis of the *Snn3-B1* Physical Region

To investigate gene content at the *Snn3-B1* locus on chromosome 5B, the sequences containing the most significant SNPs identified in the AM panel (-log_10_*P* > 16, seven SNPs) and the MAGIC population by SAM (-log_10_*P* > 16, effect > 1, five SNPs) were aligned to the IWGSC RefSeq v1.0 wheat reference genome. The seven SNPs from the AM panel delineated a physical region of 326 kb (∼6.648–6.975 Mb), while the five MAGIC SNPs delineated physical regions of 6.2 kb (6.648–6.654 Mb), located within the physical interval as defined in the AM panel. The 326-kb region was predicted to contain seven gene models, representing three high-confidence and four low-confidence gene models (Supplementary Table S6). Two markers lie within a gene model encoding a ubiquitin-conjugating enzyme: Excalibur_c47452_183 is in the 5′ untranslated region, while BobWhite_c4838_58 is a synonymous SNP located within exon 6. Three SNPs lie within gene model TraesCS5B01G005000, a P-loop containing nucleoside triphosphate hydrolases superfamily protein: GENE-3324_338 is located in intron 2, BS00091518_51 results in a synonymous substitution in exon 3, and BS00091519_51 is predicted to result in a G→D substitution at amino acid residue 675 (G675/D), outside of the 50s ribosome-binding GTPase domain. Finally, two SNPs are located in gene model TraesCS5B01G005600, encoding a putative transmembrane protein: BS00064297_51b and BS00064298_51b both represent non-synonymous mutations (L474/P and Q480/R, respectively), neither of which are predicted to lie within a known protein domain.

## Discussion

### Effector Sensitivity in European Wheat Germplasm and Its Relevance to SNB

Septoria nodorum blotch is a major disease of wheat in many growing areas, with field resistance based on multiple minor effect genes. Identification of necrotrophic effectors in *P. nodorum* provided resources with which to dissect host resistance into its constituent parts (Cockram et al., 2015) and study their interactions (Phan et al., 2016). Here we used effector screening to determine sensitivities in 480 predominantly British winter wheat varieties. The frequency of SnTox1 sensitive varieties was 28%, broadly comparable to that found for Scandinavian varieties (12%, Ruud et al., 2017a) and global collections (16%, Shi et al., 2016a), but contrasts notably against a recent screen of Australian varieties (72%, Tan et al., 2014). SnTox3 sensitivity frequency here (42%) was similar to that reported in Scandinavian germplasm (55%, Ruud et al., 2017a). Sensitivity to SnToxA was 10% in the predominantly British winter wheat germplasm collection screened here, which is notably lower than that reported in other wheat germplasm collections, e.g., 45% in Scandinavian varieties (Ruud et al., 2017a) and 65% in Western Australian wheat (Waters et al., 2011). SnToxA sensitivity was found to be present at a relatively high frequency in the spring wheat varieties in our panel. The spring and winter wheat breeding pools are relatively separate, possibly explaining the observed frequency differences in SnToxA sensitive and insensitive alleles between the two groups. Indeed, where sensitive alleles appear in the winter genepool, this can often be tracked via the parentage of the genetically spring cultivar Cadenza, a prominent variety in the British pedigree. *Tsn1* is located on the long arm of chromosome 5B, <30 Mb from the vernalization gene *VRN-B1*, a major gene influencing winter or spring growth habit (Cockram et al., 2007). Therefore, it is likely that the partitioning of *Tsn1* alleles is influenced by linkage to winter or spring alleles at *VRN-B1*.

### Genetic Mapping of SnTox3 Sensitivity

Previous studies have mapped *Snn3-B1* to the short arm of chromosome 5B (Friesen et al., 2008; Phan et al., 2016; Shi et al., 2016a; Ruud et al., 2017b). Here, genetic analysis in the AM and MAGIC populations identified *Snn3-B1* as representing the most significant genetic determinant of SnTox3 sensitivity in British germplasm, with the highly significant markers (-log_10_*P* > 10) delimiting a 327-kb interval containing seven genes. This was achieved without the need to develop populations specifically to investigate SnTox3 sensitivity, demonstrating the efficacy of using genetic resources such as AM panels and MAGIC populations for rapid genetic dissection of target traits (Cockram and Mackay, 2018). The relatively small genetic interval determined here will allow reverse genetic approaches such as genome editing and Targeting Induced Local Lesions IN Genomes (TILLING, McCallum et al., 2000) to be undertaken to help identify the gene underlying *Snn3-B1*. However, it should be noted that given we found the cultivar Chinese Spring from which the wheat reference genome sequence is derived to be insensitive to SnTox3, it is possible that the gene underlying *Snn3-B1* is deleted or degraded to such an extent that it is not predicted as a gene model. Indeed, this was the case for the gene underlying the SnToxA sensitivity locus *Tsn1*, which was found to be absent in insensitive varieties, including Chinese Spring (Faris et al., 2010).

Minor QTL for SnTox3 sensitivity have only previously been reported on chromosome 4B (Phan et al., 2016). The six QTL identified here in the MAGIC population, on chromosomes 2A, 2B, 3B, 4D, 6A, and 7B, are therefore novel. When these are compared with known QTL for SNB field resistance and *P. nodorum* juvenile resistance studies, possible overlap can be identified for the 2A MAGIC QTL *QTox3.niab-2A.1*. This likely corresponds to *Qsnb.cur-2AS.1* (Supplementary Table S7), controlling seedling SNB sensitivity using *P. nodorum* isolate SN15, as well as knock-out strains of this isolate lacking SnTox1 (*tox1–6*), a triple knock-out strain lacking SnToxA, SnTox1, and SnTox3 (*toxa13*), and seedling inoculation with culture filtrate from isolate *toxa13* (Phan et al., 2016), and to the SNB QTL identified in the Arina × Forno population via common marker gwm372 (Abeysekara et al., 2009). Similarly, the SnTox3 sensitivity QTL identified here on chromosome 3B (*QTox3.niab-3B.1*) appears to correspond to a QTL for adult plant SNB resistance in the wheat SHA3/CBRD x Naxos grown under field conditions in Norway (Ruud et al., 2017b). The creation of near isogenic lines for such QTL would allow further characterization of their effects, and potentially, isolation of their underlying genes. The MAGIC population consists of inbred lines genotyped at the F_5_ generation, with each line expected to contain ∼2% heterozygosity, allowing development of heterogeneous inbred families (HIFs) to rapidly create near isogenic lines through selfing (Tuinstra et al., 1997). As MAGIC F_5_ lines heterozygous across each of the minor QTL are available, it should now be possible to rapidly create precise genetic materials with which to investigate their effects in isolation. We note that while MAGIC QTL analysis using CIM allowed minor QTL to be detected, in comparison to MAGIC SMA analyses (and GWAS analysis in the AM panel), it did not accurately locate *Snn3-B1*. This is due to a 5BS/7BS translocation (Badaeva et al., 2007) that is known to segregate in the MAGIC population, with the resulting segregation distortion preventing genetic mapping of markers close to the translocation breakpoint (Gardner et al., 2016). As *Snn3-B1* is close to this breakpoint, the absence of the most closely linked SNPs in the genetic map prevents accurate mapping via CIM. In contrast, SMA analysis in the MAGIC population does not require markers to be genetically mapped, highlighting the importance of using both analysis methods when undertaking QTL analysis. Indeed, in addition to *Snn3-B1*, SMA and CIM both identified additional QTL, two of which were shared and two or three of which were private to each analysis method.

### Analysis of the *Snn3-B1* Physical Region

The physical region, as defined by the most significant SNPs identified in the AM panel and MAGIC population, was predicted to contain seven gene models. Gene model TraesCS5B01G005000 (containing SNPs GENE-3324_338, BS00091518_51, and BS00091519_51) is similar to *YELLOW LEAF 1/BRASSINAZOLE INSENSITIVE PALE GREEN 2* (*BPG2*), involved in the accumulation of chloroplast proteins and the salt stress response pathway in Arabidopsis (Li et al., 2016; Qi et al., 2016). The SNP that had the largest phenotypic effect from the MAGIC SMA analysis was located within gene model TraesCS5B01G005100, which encoded a “ubiquitin-conjugating enzyme E2”. This class of genes has been shown to regulate plant disease resistance, both positively and negatively. Examples include the U-box type E3 ubiquitin ligase, CMPG1, that regulates immunity in multiple plant species (González-Lamothe et al., 2006), SPL11, a negative regulator of cell death in rice (Zeng et al., 2004), and Plant U-box 22 (PUB22), PUB23, and PUB24, that negatively regulate PTI in Arabidopsis (Trujillo et al., 2008). Genes TraesCS5B01G005200, TraesCS5B01G005300, and TraesCS5B01G005400 all showed sequence similarity to protein kinases, a class of genes known to play a role in disease resistance (Xia, 2004). However, protein kinase domains were only predicted within the amino acid sequence of TraesCS5B01G005400. Finally, gene models TraesCS5B01G005500 and TraesCS5B01G005600 (containing SNPs BS00064297_51b and BS00064298_51b) both encode predicted transmembrane proteins, with BLASTn matches (≤7e^-66^) to single, unannotated genes in rice and brachypodium. Further work is needed to investigate whether any of these genes underlie *Snn3-B1*.

### The Use of Effector Sensitivity Loci for Wheat Research and Breeding

SnTox3 sensitivity and disease susceptibility had previously been reported to be poorly correlated (Friesen et al., 2009), only accounting for a significant portion of disease phenotype in adult plants segregating for sensitivity alleles at *Snn3-B1*, *Tsn1*, and *Snn2* – although when infected with *P. nodorum* isolates lacking *SnToxA*. This is consistent with the notion that the SnToxA–*Tsn1* interaction is epistatic to the SnTox3–*Snn3-B1* interaction (Friesen et al., 2008). However, more recently, the *Snn3-B1* locus has been identified in QTL analysis of adult plant field resistance to SNB in northwestern Europe (Waters et al., 2011; Lu and Lillemo, 2014; Ruud et al., 2017b). It is thought that *SnTox1* expression inhibits the transcription of *SnTox3* (Phan et al., 2016). This may explain the reason that while gene-for-gene interactions are readily identified via effector infiltration, their interactions are not always additive. However, recent work has found infiltration of wheat seedlings with culture filtrate using SnTox3 positive *P. nodorum* isolates resulted in a necrotic phenotype on wheat containing *Snn3-B1*, irrespective of the presence of *SnTox1* in the pathogen (Ruud et al., 2017b). Nevertheless, the differing associations between disease susceptibility and effector sensitivity will likely depend on the effectors present in regional pathogen populations, the interactions between these effectors, and the alleles present at their corresponding host sensitivity loci. To allow rapid selection for allelic state at *Snn3-B1*, we develop a co-dominant KASP genetic marker closely linked to the locus for use within wheat breeding programs. The marker represents a useful tool for marker-assisted selection for SNB, given the proven association between *Snn3-B1* and SNB resistance and that the marker is able to robustly call alleles. Despite the relatively simple Mendelian control of the trait in bread wheat, however, this marker is not a perfect predictor of SnTox3 sensitivity in the 457 phenotyped accessions in the AM panel – most notably, the 43 highly SnTox3 sensitive varieties that carry A:A alleles at SNP Excalibur_c47452_183 (**Table 3** and Supplementary Table S1). This is similar to the results of other studies that have attempted to identify diagnostic markers for SnTox3 sensitivity (Shi et al., 2016a; Phan et al., 2018). This observation could be due to a number of reasons, including one or a combination of the following: insufficient marker saturation, multiple alleles at the *Snn3-B1* locus, control by copy number variation, or the effect of minor QTL. Indeed, SnTox3 sensitivity in the AM panel shows more of a quantitative distribution, in contrast to the qualitative phenotypic distribution found for SnToxA sensitivity (**Figure 1**). Nevertheless, the KASP marker for SNP Excalibur_c47452_183 developed here will be of use in tracking SnTox3 sensitivity alleles where the sensitivity of the founders is known (e.g., Supplementary Table S1), and to help further narrow the *Snn3-B1* genetic interval. For example, given that nearly all varieties phenotyped that carry G:G alleles, with few exceptions, are highly sensitive (score > 3) for SnTox3, this marker could be used to remove the majority of highly sensitive varieties from a breeding program.

## Author Contributions

RD, LB, GM, and GR undertook research. RD, KG, NG, CCM, IM, and JC analyzed the data. EF, JC, KG, HP, IM, and RO provided scientific input and project resources. JC, K-CT, NG, and RO provided supervision and project management. RD and JC wrote the manuscript. All authors reviewed the manuscript.

## Conflict of Interest Statement

The authors declare that the research was conducted in the absence of any commercial or financial relationships that could be construed as a potential conflict of interest. The reviewer AT and handling Editor declared their shared affiliation.

## Footnotes

1 cran.r-project.org

2 http://lgcgenomics.com/

## Abbreviations

AM: association mapping
CIM: composite interval mapping
GWAS: genome-wide association scans
MAGIC: multi-parent advanced generation inter-cross
QTL: quantitative trait locus/loci
SMA: single marker analysis
SNB: septoria nodorum blotch
SNP: single nucleotide polymorphism
