# Supplementary material for: Assessing European Wheat Sensitivities to Parastagonospora nodorum Necrotrophic Effectors and Fine-Mapping the Snn3-B1 Locus Conferring Sensitivity to the Effector SnTox3
Source: Front Plant Sci. 2018 Jul 4;9:881. doi: 10.3389/fpls.2018.00881 (PMC6039772; doi:10.3389/fpls.2018.00881)
Supplement: Supplementary file 1 [file Table_1.pdf]

Supplementary Table 1. Association mapping panel SnToxA, SnTox1 and SnTox3 phenotypic data (mean of 3-4 replicates per variety). Score 0 = insensitive, score 4 = highly sensitive. SGH = seasonal growth habit (S=spring, W=winter. [1]Our phenotype data. [2]Source = European Wheat Database, http://genbank.vurv.cz/ewdb/. [3]Other online sources). NA = missing data. Year = consensus varietal release date, based on the information listed in column 'Year source'. BR control = brown rust control variety. [†]Listed UK = first year on UK National List; trial UK = first year in trial for UK Recommended List; genebank cz = year listed in the European Wheat Database, http://genbank.vurv.cz/ewdb/.

Ordered Variety	SGH	Origin	Year	Year source[†]	SnToxA	SnTox1	SnTox3
S1117_RAFFLES	S	GBR	1997	listed uk	4.00	0.50	4.00
S0117_MARIS_HALBERD	S	GBR	1972	genbank cz	2.00	3.25	4.00
S1591_AC_BARRIE	S	CAN	2003	listed uk	2.33	4.00	4.00
S0017_MARIS_ENSIGN	S	GBR	1968	genbank cz	0.00	4.00	4.00
S0185_SICCO	S	NLD	1973	genbank cz	4.00	4.00	4.00
S0248_HIGHBURY	S	GBR	1968	genbank cz	0.00	2.00	4.00
S0249_AINTREE	S	GBR	1981	genbank cz	1.50	2.00	4.00
S0337_SANDOWN	S	GBR	1980	genbank cz	2.33	4.00	0.00
S0406_NEWMARKET	S	GBR	NA	genbank cz	4.00	3.75	0.00
S0455_HAYDOCK	S	GBR	NA	genbank cz	3.50	3.00	0.00
S0494_SOLITAIRE	S	GBR	1985	genbank cz	1.00	4.00	1.00
S0497_JERICO	S	FRA	1983	listed uk	4.00	1.00	4.00
S0052_MARIS_DOVE	S	GBR	1971	genbank cz	4.00	2.50	4.00
S0053_MARIS_BUTLER	S	GBR	1972	genbank cz	1.25	0.25	4.00
S0547_WEMBLEY	S	GBR	1984	listed uk	3.25	0.00	0.25
S0919_CHABLIS	S	GBR	1994	listed uk	0.00	2.00	2.00
S0920_SHIRAZ	S	GBR	1994	listed uk	4.00	3.00	4.00
U100100_ANGLIA	W[1]	GBR	1987	genbank cz	0.00	3.50	1.50
U100136_ARINA	W[3]	CHE	1981	genbank cz	0.00	2.50	3.50
U100273_CAPELLE_DESPREZ	W	FRA	1946	genbank cz	NA	NA	NA
U100347_COURTOT	S[1]	FRA	1974	genbank cz	4.00	3.00	1.00
U100460_ETOILE_DE_CHOISY	W[1,2]	FRA	1950	genbank cz	1.00	4.00	2.50
U100546_GARNET	S[1]	CAN	1926	genbank cz	4.00	4.00	4.00
U100578_GREIF	W[1,2]	DEU	1989	genbank cz	0.50	3.75	2.25
U100825_MONOPOL	W[1,2,3]	DEU	1975	genbank cz	2.00	3.00	4.00
U100874_OKAPI	W[3]	DEU	1978	genbank cz	0.00	3.00	2.00
U101082_SELKIRK	S[1]	CAN	1953	genbank cz	NA	NA	NA
U101237_VIKING	W[1,2,3]	DNK	1956	genbank cz	2.00	4.00	3.00
U101246_VUKA	W[1,2,3]	DEU	1975	genbank cz	NA	NA	NA
U101415_IBIS	W[3]	DEU	1962	genbank cz	0.00	3.25	4.00
U112030_TAMBOR	W[1]	DEU	1995	genbank cz	0.00	4.00	1.75
U112495_ISENGRAIN	W[3]	FRA	1997	genbank cz	4.00	1.67	4.00
U112544_AMAROK	W[3]	FRA	NA	genbank cz	4.00	3.50	4.00
U112547_ARONDE	S[1]	FRA	1962	genbank cz	0.00	3.50	4.00
U112943_LORRAINE	W[1]	FRA	1998	genbank cz	0.00	0.67	0.25
U113096_BOSTON	W[1]	NLD	NA	NA	0.00	0.25	2.50
U9000_80_30_VERSAILLES	W[3]	FRA	NA	NA	4.00	3.75	3.50
U9001_BERSEE	W[1,2,3]	FRA	1936	genbank cz	1.13	4.00	1.13
U9002_BILBO	W[1]	GBR	1972	genbank cz	0.25	0.00	0.33

U9003_CARSTENS_V	W[3]	DEU	1921	genbank cz	NA	NA	NA
U9004_CARSTENS_VIII	W[1]	DEU	1952	genbank cz	0.00	0.75	4.00
U9005_CLEO	W[1,3]	NLD	1961	genbank cz	0.00	2.00	1.50
U9006_DW929620_511	NA	GBR	NA	NA	4.00	3.50	4.00
U9007_DW930861_509	NA	GBR	NA	NA	0.00	2.00	1.00
U9008_ELYSEE	S[1]	FRA	1968	genbank cz	0.00	0.00	1.00
U9010_HEINES_110	W[1,2]	DEU	1969	genbank cz	4.00	3.25	3.25
U9011_HEINES_PEKO	S[1]	DEU	1946	genbank cz	NA	NA	NA
U9012_HOLDFAST	W[1,2,3]	GBR	1936	genbank cz	1.50	0.00	3.99
U9013_HYBRID_46	W[1,3]	FRA	1936	genbank cz	0.00	2.25	1.00
U9014_HYBRIDE_DU_JONCQUOIS	W[1]	FRA	1933	genbank cz	0.00	4.00	0.75
U9015_JUFY_I	S[1]	BEL	1954	genbank cz	0.25	2.75	3.50
U9016_KOGA_1	S[1,3]	DEU	NA	NA	4.00	2.00	2.00
U9017_KOGA_II	S[1]	DEU	NA	NA	0.00	2.00	3.75
U9018_KRANICH	W[1,2]	DEU	1969	genbank cz	1.00	2.00	4.00
U9019_KRONJUWEL	W[1,2]	DEU	1981	genbank cz	1.00	0.00	0.50
U9020_MARNE_DESPREZ	W[1,2]	FRA	1954	genbank cz	NA	NA	NA
U9021_MINISTER	W[1,2]	BEL	1947	genbank cz	0.25	3.75	1.25
U9022_NORD_DESPREZ	W[1,3]	FRA	1945	genbank cz	0.00	0.75	0.00
U9023_NSL_92_5719	NA	GBR	NA	NA	0.00	0.00	3.00
U9024_NSL_93_5372	NA	GBR	NA	NA	0.00	0.00	1.25
U9025_NSL_94_5130	NA	GBR	NA	NA	0.33	0.00	1.33
U9026_NSL_94_6897	NA	GBR	NA	NA	0.00	0.00	0.25
U9027_NSL_WW13	NA	GBR	NA	NA	0.25	2.25	0.00
U9028_OBELISK	W[3]	NLD	1985	genbank cz	1.25	2.75	4.00
U9029_POROS	W[2]	DEU	1966	genbank cz	0.75	2.00	2.00
U9030_PROFESSEUR_DELOS	W[1,2]	BEL	1937	genbank cz	1.00	NA	NA
U9031_PROFESSEUR_MARCHAL	W[1,2,3]	BEL	1957	genbank cz	1.50	NA	NA
U9032_RIEBESEL_57/41	W[1]	DEU	NA	NA	4.00	1.00	4.00
U9033_STELLA	W[1]	BEL	1957	genbank cz	0.00	1.00	2.25
U9034_TADEPI	W[1,3]	FRA	1949	genbank cz	0.00	2.50	0.00
U9035_TADORNA	W[2,3]	NLD	1966	genbank cz	0.00	2.50	2.00
U9036_THATCHER	S[1,2]	USA	1934	genbank cz	NA	NA	NA
U9038_VILMORIN_23	W[1,2]	FRA	1923	genbank cz	NA	NA	NA
U9039_VILMORIN_27	W[3]	FRA	1928	genbank cz	NA	NA	NA
U9040_VILMORIN_29	W[1,2]	FRA	1929	genbank cz	0.50	2.25	NA
U9042_WH929623_5	NA	NA	NA	NA	0.00	3.50	1.00
U9043_YEOMAN	W[3]	FRA	1916	genbank cz	0.00	2.75	3.25
W0010_MILDRESS	W	NLD	1964	trial uk	1.00	3.33	3.50
W1003_VIVANT	W	GBR	1990	BR ref	4.00	4.00	4.00
W1008_JACADI	W	FRA	1997	listed uk	0.25	4.00	4.00
W1014_BANKER	W	GBR	1996	listed uk	0.25	4.00	1.25
W1019_TEMPLE	W	NA	1996	listed uk	0.00	3.75	0.00
W1020_TILBURI	W	FRA	1994	BR ref	4.00	3.50	2.75
W1028_TEMPEST	W	NA	1997	listed uk	0.00	2.50	2.75
W1029_BLAZE	W	GBR	1996	listed uk	0.00	2.00	4.00
W0103_MARIS_FREEMAN	W	GBR	1970	trial uk	0.00	2.25	0.25
W1030_CANTATA	W	GBR	1996	listed uk	0.00	1.50	0.00
W1031_FALSTAFF	W	GBR	1997	listed uk	0.00	4.00	4.00
W1033_SAVANNAH	W	GBR	1996	listed uk	0.00	1.67	1.50
W1035_MAVERICK	W	GBR	1996	listed uk	0.00	0.33	4.00
W1039_CHAUCER	W	GBR	1996	listed uk	0.00	0.00	0.67

W1040_WESTON	W	GBR	1996	listed uk	0.00	2.75	0.67
W1046_OBERON	W	GBR	1996	listed uk	0.00	2.75	2.00
W1047_KRAKATOA	W	GBR	1996	listed uk	0.00	2.50	3.75
W1048_WELLINGTON	W	GBR	1997	listed uk	1.50	1.75	0.00
W0105_MARIS_MARKSMAN	W	GBR	1975	trial uk	0.25	1.25	2.33
W1062_SHANGO	W	GBR	1995	trial uk	0.00	2.75	1.00
W1070_CLAIRE	W	GBR	1997	listed uk	0.50	2.15	1.01
W1078_MARSHAL	W	GBR	1997	listed uk	0.00	3.00	3.25
W1088_ROSETTE	W	NA	1997	listed uk	0.25	0.75	4.00
W0109_MEGA	W	GBR	1970	trial uk	0.00	4.00	0.00
W1092_SHAMROCK	W	FRA	1997	listed uk	0.00	4.00	4.00
W0110_CARIBO	W	DEU	1968	genbank cz	0.75	0.00	1.25
W1100_BUCHAN	W	GBR	1997	listed uk	NA	NA	NA
W1108_DATUM	W	GBR	1999	listed uk	0.00	2.00	3.00
W0111_ATOU	W	FRA	1970	trial uk	0.00	3.50	4.00
W1111_AARDVARK	W	GBR	1997	listed uk	0.00	0.00	1.75
W1115_DICKINS	W	NA	1998	listed uk	4.00	0.00	2.00
W1120_FLAIR	W	DEU	1996	BR ref	0.00	0.00	0.00
W1122_ATOLL*	W	FRA	1996	BR ref	0.00	0.00	1.00
W1126_ECLIPSE	W	GBR	1999	listed uk	0.00	0.00	4.00
W1133_AGAMI	W	GBR	1996	BR ref	0.00	0.00	2.75
W1137_BROILER	W	GBR	1999	listed uk	0.00	0.25	0.00
W1147_NAPIER	W	GBR	1998	listed uk	0.00	0.00	1.00
W1149_WICKHAM	W	GBR	1998	listed uk	0.00	0.00	0.00
W1151_CHICAGO	W	GBR	1998	listed uk	0.00	0.00	3.50
W1152_EXPLOSIV	W	GBR	1999	listed uk	0.00	0.00	0.00
W1153_GENGHIS	W	GBR	1999	listed uk	0.50	3.50	4.00
W1155_DENVER*	W	GBR	1998	listed uk	0.00	2.00	2.50
W1156_BRANDO	W	GBR	1999	listed uk	0.00	4.00	4.00
W1157_RANGER	W	GBR	1998	listed uk	0.75	3.50	3.33
W1161_DERWENT*	W	USA	1999	listed uk	0.00	3.00	4.00
W1169_MEXICO	W	NA	1998	listed uk	0.00	2.00	2.75
W1184_ARLINGTON	W	USA	2000	listed uk	0.00	2.75	3.50
W1195_VERDON	W	FRA	1999	listed uk	1.00	2.00	2.33
W1196_ELECTRON	W	FRA	2000	listed uk	0.00	0.67	4.00
W1197_DORIAL	W	FRA	1999	listed uk	0.00	0.00	0.00
W1199_MILESTONE	W	GBR	1999	listed uk	NA	NA	NA
W1200_OPTION	W	GBR	1999	listed uk	0.00	1.50	2.00
W1201_REYDON	W	GBR	1999	listed uk	0.00	2.25	2.00
W1202_OXBOW	W	GBR	1999	listed uk	0.00	1.25	1.00
W1204_SLADE	W	GBR	1999	listed uk	0.00	2.50	2.33
W1205_POTENT	W	GBR	1999	listed uk	0.00	2.00	3.75
W1206_GOLDLACE	W	GBR	1999	listed uk	0.50	2.50	4.00
W1210_ORTON	W	GBR	2000	listed uk	0.00	0.50	0.00
W1211_ODYSSEY	W	GBR	1999	listed uk	0.00	1.75	3.50
W1213_EXSEPT	W	DEU	1999	listed uk	1.25	3.50	4.00
W1220_DEBEN	W	GBR	1999	listed uk	0.00	2.25	2.00
W1222_ARRIVA	W	GBR	1999	listed uk	0.25	4.00	1.75
W1223_CANTERBURY	W	GBR	1999	listed uk	0.00	0.50	0.00
W1224_CYBER	W	GBR	1997	BR ref	4.00	2.50	4.00
W1226_TRAVIX	W	GBR	2000	listed uk	0.00	2.50	4.00
W1227_BISCAY	W	GBR	1999	listed uk	0.00	0.00	1.67
W1233_ARK	W	DEU	2000	listed uk	0.00	0.00	0.67

W1235_EPOCH	W	NA	1997	BR ref	0.00	1.50	4.00
W1240_NEXUS	W	NA	1997	BR ref	0.00	0.00	0.00
W1249_CHEQUER	W	GBR	1998	BR ref	0.00	1.25	0.67
W1251_HARROW	W	GBR	1998	BR ref	0.25	1.50	2.50
W1257_RICHMOND	W	GBR	2000	listed uk	0.33	1.50	1.75
W1258_CHATSWORTH	W	NLD	2000	listed uk	0.00	0.00	0.33
W1259_WOBURN	W	NLD	2000	listed uk	1.00	3.25	4.00
W1262_MACRO	W	GBR	2000	listed uk	NA	2.00	2.50
W1263_FENDER	W	GBR	2001	listed uk	1.50	2.75	2.75
W1266_ACCESS	W	GBR	2000	listed uk	NA	2.75	3.50
W1267_ANGLO	W	GBR	2000	listed uk	NA	2.75	2.50
W1269_POSIT	W	GBR	2000	listed uk	2.00	4.00	4.00
W1270_COMET	W	GBR	1998	BR ref	2.00	3.75	4.00
W1271_FEAST	W	GBR	2000	listed uk	1.75	4.00	4.00
W1272_RAMPART	W	GBR	2000	listed uk	1.33	2.50	4.00
W1273_VAULT	W	GBR	1998	BR ref	1.00	2.00	2.67
W1275_KEMPT	W	GBR	1998	BR ref	0.00	4.00	4.00
W1276_ALCHEMIST	W	GBR	2000	listed uk	NA	4.00	4.00
W1277_FRELON	W	GBR	2001	listed uk	4.00	2.00	0.00
W1278_PHLEBAS	W	GBR	2000	listed uk	0.00	3.75	0.00
W1280_A13_98	W	DNK	NA	NA	0.00	2.00	4.00
W1281_XI19	W	NLD	2000	listed uk	4.00	3.87	4.00
W1282_SOLSTICE*	W	NLD	2001	listed uk	3.75	1.75	4.00
W1286_STORM	W	NLD	2000	listed uk	0.00	2.00	4.00
W1292_VIRTUOSE	W	FRA	1998	BR ref	0.00	1.00	4.00
W1295_SABRE*	W	GBR	2000	listed uk	0.00	0.00	0.50
W1300_WINDSOR	W	DEU	1998	BR ref	0.00	2.25	4.00
W1310_CAPNOR	W	FRA	1999	BR ref	0.00	0.50	4.00
W1321_PR21R60	W	NA	2001	listed uk	0.00	1.00	4.00
W1326_TELLUS	W	GBR	2002	listed uk	0.00	2.75	4.00
W1327_BRUNEL	W	GBR	2001	listed uk	0.00	2.00	4.00
W1328_WIZARD***	W	GBR	2002	listed uk	0.00	0.00	0.50
W1329_ZAKA	W	GBR	2002	listed uk	0.00	2.00	2.75
W0133_TALENT	W	FRA	1973	genbank cz	0.00	0.00	0.00
W1330_ROBIGUS	W	GBR	2002	listed uk	0.00	1.03	1.50
W1331_CONTEXT	W	GBR	2001	listed uk	0.00	2.33	2.00
W1332_CONVOY	W	GBR	2001	listed uk	0.00	4.00	4.00
W1333_ASHANTI	W	GBR	2001	listed uk	0.00	1.67	0.00
W1335_SCORPION_25	W	GBR	2001	listed uk	4.00	4.00	4.00
W1336_WARLOCK_24	W	GBR	2001	listed uk	4.00	4.00	4.00
W0134_CLEMENT	W	NLD	1971	trial uk	0.00	1.00	2.00
W1343_CARLTON	W	GBR	2001	listed uk	4.00	0.67	3.00
W1353_GOODWILL	W	GBR	2001	listed uk	0.00	2.50	3.00
W1359_INSIGHT	W	GBR	2001	listed uk	0.00	2.00	1.75
W1361_MALLET	W	GBR	2001	listed uk	0.00	0.50	3.00
W1364_RIVET	W	GBR	2001	listed uk	0.00	1.50	0.50
W1367_FIELDER	W	GBR	2001	listed uk	0.00	1.00	4.00
W1370_HARBOUR	W	GBR	2001	listed uk	0.00	1.67	NA
W1376_EINSTEIN	W	GBR	2002	listed uk	0.00	0.75	2.00
W1380_ARRAN	W	GBR	2002	listed uk	0.00	1.33	1.00
W1383_CHARDONNAY	W	GBR	2000	trial uk	0.00	0.25	2.67
W1388_CORDIALE	W	GBR	2003	listed uk	0.00	3.63	0.50
W1392_QUEST	W	GBR	2003	listed uk	0.25	1.25	0.25

W1395_SENATOR	W	GBR	2003	listed uk	0.67	2.67	2.25
W1400_FLAXEN	W	GBR	2002	listed uk	0.00	1.50	0.67
W1407_AWARD	W	DEU	2002	listed uk	0.25	0.67	2.00
W1409_MAYFAIR	W	NA	2002	listed uk	0.00	2.25	NA
W1411_DICKSON	W	NA	2002	listed uk	0.00	2.25	1.25
W1414_VECTOR	W	GBR	2002	listed uk	0.00	0.33	3.33
W1415_SMUGGLER	W	GBR	2002	listed uk	0.00	2.67	NA
W1426_ISTABRAQ	W	GBR	2003	listed uk	0.00	2.67	1.50
W1427_NIJINSKY	W	GBR	2003	listed uk	0.00	2.75	0.25
W1428_SANCERRE	W	NA	2003	listed uk	0.00	1.75	0.00
W1429_ALSACE	W	FRA	2002	listed uk	0.25	2.00	2.75
W1435_SW_TATAROS	W	DEU	2003	listed uk	1.50	4.00	1.00
W1438_CAPHORN	W	GBR	2000	BR ref	0.00	4.00	4.00
W1439_DART*	W	GBR	2002	listed uk	0.00	1.25	1.33
W1441_MONUMENT	W	GBR	2002	listed uk	0.00	2.00	2.00
W1442_GLADIATOR*	W	GBR	2002	listed uk	0.00	2.28	4.00
W1443_HERITAGE	W	GBR	2002	listed uk	0.00	2.00	3.25
W1444_M007	W	NA	2002	listed uk	0.00	3.25	4.00
W1445_BELTER	W	GBR	2002	listed uk	0.25	2.00	2.00
W1447_BENTLEY	W	FRA	2002	listed uk	NA	NA	NA
W1449_WELFORD	W	GBR	2002	listed uk	0.00	1.67	3.50
W1450_PENNANT*	W	GBR	2002	listed uk	0.00	0.00	2.00
W1456_RAGLAN	W	GBR	2003	listed uk	0.00	1.33	3.25
W1461_MAYFIELD	W	GBR	2003	listed uk	0.00	1.75	4.00
W1462_AMBROSIA	W	GBR	2003	listed uk	0.00	0.00	0.00
W1463_EQUATOR	W	GBR	2003	listed uk	0.00	2.00	3.00
W1464_EXTEND	W	GBR	2003	listed uk	0.00	1.75	1.00
W1467_MAGNITUDE	W	GBR	2003	listed uk	0.00	1.25	3.25
W1468_DEFENDER	W	GBR	2003	listed uk	0.00	2.50	3.00
W1469_SCANDIA	W	GBR	2003	listed uk	0.00	4.00	3.25
W1470_CHOICE*	W	GBR	2003	listed uk	0.25	4.00	2.00
W1477_ATLANTA	W	GBR	2004	listed uk	0.00	0.00	0.50
W1482_GLASGOW	W	NA	2003	listed uk	0.00	2.00	4.00
W1489_ISIDOR	W	FRA	2002	listed FRA	0.00	0.50	4.00
W1497_SAMURAI	W	DEU	2003	listed uk	0.00	2.25	4.00
W1499_PREDATOR	W	GBR	2003	listed uk	0.00	3.33	3.75
W1502_BROMPTON	W	GBR	2002	trial uk	0.00	1.12	2.62
W1511_CHESTER	W	GBR	2003	listed uk	0.00	0.00	0.25
W1512_EXETER	W	FRA	2003	listed uk	0.50	1.50	0.50
W1532_MASCOT*	W	GBR	2004	listed uk	0.75	2.50	1.67
W1541_PIRANHA	W	GBR	2004	listed uk	0.25	1.25	4.00
W1542_DIRECTOR	W	GBR	2004	listed uk	1.00	1.00	2.67
W1543_ASAGAI	W	GBR	2003	trial uk	0.50	1.25	4.00
W1545_ZEBEDEE	W	GBR	2004	listed uk	1.00	1.33	2.67
W1546_GATSBY	W	GBR	2004	listed uk	0.00	2.00	4.00
W1549_FASTNET	W	GBR	2004	listed uk	0.50	4.00	2.50
W1550_DOVER	W	GBR	2005	listed uk	0.00	0.00	0.33
W1556_HOURRA	W	GBR	2004	listed uk	1.25	2.00	2.00
W1561_HYPERION	W	FRA	2004	listed uk	0.50	2.33	0.00
W1564_ALCHEMY	W	GBR	2004	listed uk	0.00	2.12	0.37
W1573_AARDEN	W	FRA	2005	listed uk	0.00	0.00	0.00
W1577_HURLEY	W	GBR	2005	listed uk	0.00	1.25	0.00
W1578_KIPLING	W	FRA	2004	listed uk	0.00	1.75	0.00

W0158_MARIS_FUNDIN	W	GBR	1972	trial uk	0.00	3.25	0.00
W1599_BATTALION	W	GBR	2005	listed uk	0.00	4.00	1.00
W1606_OCHRE	W	GBR	2003	BR ref	0.00	0.50	0.00
W1611_CONTENDER	W	GBR	2006	listed uk	0.00	0.00	4.00
W1621_GULLIVER	W	GBR	2005	listed uk	0.00	2.00	4.00
W1625_BENEDICT	W	GBR	2006	listed uk	0.00	3.00	1.67
W1649_SAHARA	W	NLD	2005	listed uk	0.00	4.00	1.75
W1652_HUMBER	W	GBR	2005	listed uk	0.00	4.00	1.00
W1658_OAKLEY	W	GBR	2006	listed uk	1.50	2.25	4.00
W1666_CANADAIR	W	NA	2006	listed uk	1.33	4.00	0.00
W1667_BUZZER	W	GBR	2006	listed uk	0.33	4.00	4.00
W1668_MAXWELL	W	NA	2006	listed uk	0.00	0.67	0.00
W1677_ROCKY*	W	GBR	2005	trial uk	0.00	3.25	2.75
W1680_MUSKETEER	W	GBR	2005	trial uk	0.00	3.75	0.50
W1687_MARKSMAN**	W	GBR	2006	listed uk	0.00	4.00	2.50
W0169_ALCEDO	W	DEU	1974	genbank cz	4.00	3.25	0.50
W1695_BOWINDO	W	GBR	2007	listed uk	0.00	4.00	2.00
W1704_VELOCITY	W	GBR	2006	listed uk	3.00	2.50	4.00
W0171_HARDI	W	NA	1969	genbank cz	0.67	3.00	0.00
W1714_ORATOR	W	FRA	2007	listed uk	0.00	3.00	4.00
W1724_EMERALD	W	GBR	2007	listed uk	1.00	4.00	0.67
W1725_DUXFORD	W	GBR	2006	listed uk	3.25	4.00	4.00
W1726_LIMERICK	W	GBR	2006	listed uk	4.00	3.25	4.00
W1727_MONTY	W	GBR	2006	listed uk	0.00	3.25	2.00
W1728_ZANATAN	W	GBR	2006	listed uk	0.25	4.00	1.50
W0173_FLANDERS	W	FRA	1973	trial uk	1.50	NA	4.00
W1731_HEREFORD	W	DNK	2005	trial uk	0.00	4.00	0.33
W1737_JB_DIEGO	W	GBR	2006	listed uk	0.00	3.00	1.00
W1746_WALPOLE	W	NLD	2005	BR ref	4.00	2.00	4.00
W1753_BATSMAN	W	NA	2005	BR ref	0.00	2.00	4.00
W1756_LANGDALE	W	GBR	2005	BR ref	0.00	1.75	4.00
W1760_GALTIC	W	FRA	2005	BR ref	0.00	4.00	4.00
W1766_GALLANT	W	GBR	2005	BR ref	0.00	2.75	0.75
W1769_SHOGUN	W	GBR	2005	BR ref	0.00	0.00	2.00
W1779_CELEBRATION	W	NA	2005	BR ref	0.00	0.75	1.25
W0178_KINSMAN	W	GBR	1973	trial uk	0.00	3.75	4.00
W1787_SCOUT	W	GBR	2005	BR ref	1.00	0.00	1.00
W1789_QPLUS	W	NA	2005	BR ref	0.25	0.00	4.00
W0179_HOBBIT	W	GBR	1973	trial uk	1.50	0.00	2.33
W1790_LEAR	W	GBR	2005	BR ref	1.25	0.75	3.50
W1795_BANTAM*	W	GBR	2005	BR ref	4.00	2.00	4.00
W1798_CASSIUS	W	GBR	2005	BR ref	0.25	0.25	1.25
W0018_MARIS_RANGER	W	GBR	1966	trial uk	2.00	4.00	2.75
W1801_PANORAMA	W	GBR	2005	BR ref	4.00	2.00	4.00
W1806_TIMARU	W	GBR	2005	BR ref	0.00	0.00	4.00
W1808_ACROBAT	W	GBR	2005	BR ref	0.00	0.00	1.75
W1811_GRAFTON	W	GBR	2005	BR ref	0.00	3.50	0.25
W1813_CONQUEROR	W	GBR	2005	BR ref	1.50	0.00	2.00
W1825_TYRELL	W	GBR	2006	BR ref	NA	NA	NA
W1827_CHEVRON**	W	GBR	2006	BR ref	0.00	3.25	4.00
W1830_ROCHFORT	W	GBR	2006	BR ref	0.00	0.00	3.50
W1853_INVICTA	W	GBR	2006	BR ref	0.00	0.00	3.25
W1858_SANTANA	W	GBR	2006	BR ref	0.00	2.00	4.00

W1860_EDMUNDS	W	GBR	2006	BR ref	0.00	0.00	0.00
W1865_WARRIOR**	W	GBR	2006	BR ref	0.00	0.00	2.67
W1871_STALWART	W	GBR	2006	BR ref	0.50	0.00	1.50
W1877_KWS_CURLEW	W	GBR	2006	BR ref	4.00	2.00	4.00
W1880_KWS_STERLING	W	GBR	2006	BR ref	0.00	1.00	1.25
W1882_HORIZON	W	GBR	2006	BR ref	0.00	0.25	1.25
W1883_KWS_QUARTZ	W	GBR	2006	BR ref	NA	NA	NA
W1885_BELUGA	W	GBR	2006	BR ref	0.00	0.67	0.75
W1892_CADOGAN	W	GBR	2007	BR ref	0.00	0.25	2.25
W1895_DENMAN	W	GBR	2007	BR ref	0.00	0.00	0.00
W1904_RAINBOW*	W	DEU	2007	BR ref	0.50	1.25	4.00
W1907_KWS_PODIUM	W	GBR	2007	BR ref	0.50	3.00	0.00
W1909_KWS_GYMNAST	W	GBR	2007	BR ref	0.00	0.50	1.25
W1911_KWS_TARGET	W	GBR	2007	BR ref	0.00	2.75	0.00
W1916_KWS_SANTIAGO	W	GBR	2007	BR ref	1.50	4.00	4.00
W0192_DURIN	W	GBR	1968	genbank cz	2.50	0.75	3.00
W1922_COCOON	W	GBR	2007	BR ref	0.50	1.75	4.00
W0193_TIPSTAFF	W	GBR	NA	NA	0.00	4.00	2.75
W1933_ORBIT	W	GBR	2007	BR ref	2.00	4.00	1.50
W1940_GRAVITAS	W	GBR	2007	BR ref	0.50	4.00	2.00
W1941_STIGG	W	GBR	2007	BR ref	0.00	1.50	0.00
W1943_LAZARUS	W	GBR	2007	BR ref	0.00	4.00	0.00
W1947_SHELDON	W	GBR	2007	BR ref	2.00	4.00	2.00
W1954_TUXEDO	W	GBR	2007	BR ref	1.00	2.25	4.00
W0196_SPORTSMAN	W	GBR	1974	trial uk	0.00	4.00	2.00
W0201_ARMADA	W	GBR	1974	trial uk	0.00	2.75	4.00
W0205_KADOR	W	GBR	1974	trial uk	0.75	2.50	3.00
W0023_TOMMY	W	FRA	1967	trial uk	0.50	2.00	1.25
W0230_HUSTLER	W	GBR	1975	trial uk	0.00	0.00	0.00
W0231_BRIGAND	W	GBR	1975	trial uk	3.25	3.00	4.00
W0233_MARDLER	W	GBR	1975	trial uk	0.00	4.00	3.33
W0235_ARGENT	W	GBR	1979	genbank cz	0.50	1.00	3.00
W0243_AQUILA	W	GBR	1975	trial uk	NA	NA	NA
W0260_ANVIL	W	GBR	1982	genbank cz	0.00	0.00	0.00
W0265_COPAIN	W	FRA	1976	trial uk	2.00	0.50	1.68
W0270_WIZARD	W	GBR	1983	genbank cz	0.00	0.00	1.75
W0271_IONA	W	GBR	1976	trial uk	1.25	0.25	2.00
W0272_SENTRY	W	GBR	1976	trial uk	0.25	0.00	1.00
W0273_VILLEIN	W	DEU	NA	NA	1.00	0.00	2.50
W0274_BOUNTY	W	GBR	1976	trial uk	0.75	0.00	3.00
W0276_VIRTUE	W	GBR	1976	trial uk	1.00	0.00	1.50
W0286_HERALD	W	GBR	NA	NA	0.25	0.00	1.75
W0287_AVALON	W	GBR	1979	trial uk	0.00	0.50	0.50
W0288_MAESTRO	W	GBR	1976	genbank cz	0.00	1.33	2.00
W0289_SHIRE	W	GBR	1979	genbank cz	0.00	1.25	1.00
W0291_HEDGEHOG	W	GBR	NA	NA	0.25	1.50	2.75
W0296_GRANTA	W	GBR	1980	genbank cz	0.00	3.33	0.67
W0311_ABELE	W	GBR	1978	trial uk	0.00	0.50	0.00
W0317_CHAMPLEIN	W	FRA	1959	trial uk	0.00	1.00	0.67
W0319_MARIS_WIDGEON	W	GBR	1960	trial uk	0.00	0.67	1.67
W0321_NORMAN	W	GBR	1979	trial uk	0.50	2.50	0.67
W0323_FLAMBEAU	W	GBR	NA	NA	0.00	2.00	0.33
W0325_RAPIER	W	GBR	1978	trial uk	0.00	3.00	3.25

W0328_DISPONENT	W	DEU	1975	genbank cz	1.00	0.50	3.00
W0364_LONGBOW	W	GBR	1979	trial uk	1.00	2.00	0.00
W0371_JENA	W	NA	1980	genbank cz	1.33	3.25	4.00
W0389_STETSON	W	GBR	1980	trial uk	0.00	2.00	NA
W0039_MARIS_SETTLER	W	GBR	1967	trial uk	0.00	3.25	0.00
W0004_BOUQUET	W	FRA	1965	trial uk	4.00	2.75	2.75
W0041_MARIS_BEACON	W	GBR	1967	trial uk	0.67	3.33	4.00
W0042_MARIS_NIMROD	W	GBR	1968	trial uk	0.50	0.67	4.00
W0423_HAMMER	W	GBR	1981	trial uk	1.33	1.33	0.00
W0043_MARIS_ENVOY	W	GBR	1974	genbank cz	0.00	NA	1.00
W0438_MITHRAS	W	GBR	1978	trial uk	2.00	2.00	0.00
W0439_AVOCET	W	GBR	NA	NA	1.75	4.00	0.00
W0440_GALAHAD	W	GBR	1982	listed uk	0.88	2.25	0.00
W0464_FRONTIER	W	GBR	1981	genbank cz	4.00	1.75	3.75
W0479_AMBASSADOR	W	GBR	1982	trial uk	0.25	3.50	4.00
W0486_MOULIN	W	GBR	1980	trial uk	0.50	2.50	3.50
W0488_RENARD	W	GBR	1982	trial uk	1.00	4.00	3.67
W0489_BROCK	W	GBR	1982	trial uk	1.67	2.25	3.50
W0491_BRIMSTONE	W	GBR	1982	trial uk	1.00	3.67	2.50
W0496_TONIC	W	GBR	1983	listed uk	4.00	1.00	2.50
W0509_BOXER	W	GBR	1982	trial uk	0.00	1.67	1.00
W0527_GAWAIN	W	GBR	1984	trial uk	4.00	2.75	4.00
W0528_ASLAN	W	GBR	1982	genbank cz	3.50	1.25	4.00
W0529_CRAFTSMAN	W	GBR	1987	genbank cz	1.00	2.25	1.00
W0531_BOOTY	W	GBR	NA	NA	0.75	1.00	4.00
W0533_MERCIA	W	GBR	1984	listed uk	0.25	0.00	0.00
W0537_SLEJPNER	W	SWE	1983	trial uk	0.82	1.00	0.00
W0544_AXONA	W	NLD	1984	listed uk	4.00	3.75	4.00
W0565_PARADE	W	GBR	1984	trial uk	0.00	1.00	1.00
W0584_SARSEN	W	GBR	1987	genbank cz	0.50	1.33	1.00
W0585_RENDEZVOUS	W	GBR	1984	trial uk	1.00	1.67	1.75
W0590_CINNABAR	W	GBR	1985	genbank cz	0.00	1.50	0.75
W0591_HORNET	W	GBR	1984	trial uk	0.00	1.67	0.33
W0607_APOLLO	W	DEU	1985	trial uk	0.00	0.75	0.67
W0610_SOLEIL	W	FRA	1986	listed uk	0.00	0.00	NA
W0612_ARMINDA	W	NLD	1976	genbank cz	2.50	2.67	0.00
W0618_MERLIN	W	DEU	1956	trial uk	0.00	4.00	3.00
W0626_SQUADRON	W	GBR	NA	NA	0.75	2.75	0.00
W0628_RIBAND	W	GBR	1987	listed uk	0.00	0.67	0.00
W0063_BENNO	W	DEU	1969	trial uk	0.00	0.00	4.00
W0635_MANDATE	W	GBR	1985	trial uk	1.25	2.25	0.00
W0065_MARIS_PLOUGHMAN	W	GBR	1972	genbank cz	0.25	1.75	1.25
W0066_MARIS_HUNTSMAN	W	GBR	1969	trial uk	0.00	1.00	0.33
W0067_MARIS_TEMPLAR	W	GBR	1969	trial uk	0.00	1.75	4.00
W0670_APOSTLE	W	GBR	1986	trial uk	1.50	1.25	3.00
W0671_PASTICHE	W	GBR	1988	listed uk	0.50	3.00	0.00
W0672_FRESCO	W	GBR	1988	listed uk	1.25	2.25	4.00
W0682_URBAN	W	DEU	1988	trial uk	1.67	1.00	4.00
W0692_BEAVER	W	GBR	1989	listed uk	0.00	3.00	2.00
W0694_HAVEN	W	GBR	1988	listed uk	0.25	3.25	4.00
W0707_AXIAL	W	FRA	1988	trial uk	2.50	2.50	3.00
W0724_EKLA	W	FRA	1988	genbank cz	1.50	3.50	3.75
W0728_TALON	W	DEU	1988	trial uk	0.50	2.50	4.00

W0732_DEAN	W	GBR	1987	trial uk	0.00	2.75	4.00
W0735_TARA	W	GBR	1988	trial uk	0.00	3.00	3.25
W0736_HEREWARD	W	GBR	1989	listed uk	1.00	2.37	4.00
W0755_ORESTIS	W	DEU	1988	genbank cz	0.00	1.50	4.00
W0756_SITKA	W	GBR	NA	NA	0.00	1.25	0.00
W0759_ADMIRAL	W	GBR	1990	listed uk	0.00	0.00	2.00
W0760_SOLDIER	W	GBR	NA	NA	0.25	1.50	2.00
W0765_SAREK	W	SWE	1992	genbank cz	4.00	2.00	4.00
W0770_LANCELOT	W	NA	2002	listed FRA	0.67	2.00	3.50
W0773_TORONTO	W	DEU	1990	listed DEU	1.00	0.00	0.75
W0775_ESTICA	W	NLD	1990	listed uk	0.00	2.00	3.00
W0776_WASP	W	GBR	1990	listed uk	2.75	1.00	4.00
W0783_CAPRIMUS	W	GBR	1994	genbank cz	0.00	0.00	4.00
W0785_ARISTOCRAT	W	GBR	1992	genbank cz	NA	2.00	4.00
W0787_TORFRIDA	W	GBR	1988	trial uk	1.00	0.50	4.00
W0798_CAMP_REMY	W	FRA	1980	listed FRA	0.00	0.00	1.00
W0801_ASTRON	W	DEU	1990	BR ref	0.00	4.00	4.00
W0803_FLETUM	W	NLD	1990	BR ref	0.00	2.50	4.00
W0806_DIABLO	W	GBR	1990	BR ref	0.75	2.00	1.33
W0808_SPARK	W	GBR	1991	listed uk	4.00	2.00	3.50
W0810_ZODIAC	W	GBR	1990	trial uk	0.00	1.75	0.67
W0811_FENDA	W	GBR	1990	BR ref	0.67	4.00	4.00
W0817_HUSSAR	W	GBR	1991	listed uk	0.50	1.50	1.25
W0818_BRIGADIER	W	GBR	1991	listed uk	1.38	0.63	2.71
W0824_BRYDEN	W	GBR	1990	BR ref	0.25	2.50	0.50
W0825_ADROIT	W	GBR	1990	BR ref	0.75	2.75	4.00
W0826_LEO*	W	NA	1990	BR ref	0.00	0.33	0.50
W0827_RENOWN	W	NA	1990	BR ref	0.50	4.00	4.00
W0828_HUNTER	W	GBR	1991	listed uk	0.00	1.67	2.67
W0829_VERITAS	W	GBR	1990	BR ref	0.00	4.00	4.00
W0833_CADENZA	W	GBR	1991	listed uk	4.00	4.00	4.00
W0834_SOISSONS	W	FRA	1990	listed uk	3.72	2.87	4.00
W0844_TREND	W	DEU	1990	BR ref	0.00	4.00	3.00
W0847_FLAME	W	GBR	1992	listed uk	0.00	3.00	0.50
W0848_WOODSTOCK	W	GBR	1992	listed uk	0.00	4.00	2.00
W0856_LYNX*	W	GBR	1992	listed uk	0.00	3.50	1.25
W0858_RIALTO	W	GBR	1992	listed uk	0.25	3.19	4.00
W0859_ANDANTE	W	GBR	1991	trial uk	0.50	1.00	1.33
W0862_ATLA	W	GBR	1990	BR ref	0.00	2.00	4.00
W0863_OSTARA	W	NA	1990	BR ref	0.00	0.00	0.00
W0867_PROPHET	W	GBR	1991	trial uk	0.00	2.50	0.00
W0878_BEAUFORT	W	GBR	1993	listed uk	0.00	2.00	0.75
W0881_ENCORE	W	GBR	1993	listed uk	4.00	2.67	2.00
W0882_CONSORT	W	GBR	1993	listed uk	0.00	2.00	2.00
W0886_TURPIN	W	GBR	1991	BR ref	0.00	2.00	0.00
W0896_DYNAMO	W	GBR	1993	listed uk	0.00	2.00	1.00
W0900_TRAWLER	W	GBR	1991	BR ref	0.00	1.00	3.00
W0931_CAXTON	W	GBR	1994	listed uk	0.00	2.00	3.75
W0932_REAPER	W	GBR	1994	listed uk	0.00	2.00	0.33
W0933_RALEIGH	W	GBR	1995	listed uk	0.00	3.00	1.75
W0934_DRAKE	W	GBR	1996	listed uk	0.00	2.00	4.00
W0938_SHANNON	W	GBR	1992	BR ref	0.00	0.75	3.75
W0939_CHARGER	W	GBR	1994	listed uk	0.00	2.75	3.33

W0940_CLOVE	W	GBR	1995	listed uk	0.00	2.25	3.75
W0942_NEWHAVEN	W	NA	1992	BR ref	0.00	1.00	2.00
W0943_GALATEA	W	GBR	1995	listed uk	0.00	1.00	0.75
W0945_MAGELLAN	W	GBR	1995	listed uk	0.33	1.50	3.75
W0946_CHIANTI	W	GBR	1995	listed uk	0.00	1.33	1.00
W0954_CROFTER	W	GBR	1994	listed uk	0.00	0.67	0.00
W0955_RITMO	W	NLD	1992	BR ref	0.00	1.75	4.00
W0966_RUBENS	W	FRA	1993	BR ref	2.00	0.00	0.00
W0967_ABBOT	W	GBR	1995	listed uk	1.33	1.75	2.75
W0970_IMPALA	W	GBR	1996	listed uk	0.50	1.00	0.50
W0972_ACCLAIM	W	GBR	1995	listed uk	0.00	2.00	2.00
W0973_MADRIGAL	W	GBR	1995	listed uk	0.50	0.67	4.00
W0978_HARRIER	W	GBR	1995	listed uk	0.00	2.50	0.50
W0980_MALACCA	W	GBR	1995	listed uk	0.50	4.00	0.00
W0982_HUDSON	W	GBR	1993	BR ref	0.00	4.00	0.00
W0983_EQUINOX	W	GBR	1995	listed uk	1.33	4.00	2.00
W0992_HOLSTER	W	GBR	1993	BR ref	0.00	2.50	4.00
W0994_WARRIOR	W	GBR	1995	listed uk	0.00	1.00	2.00
