# Supplementary material for: Assessing European Wheat Sensitivities to Parastagonospora nodorum Necrotrophic Effectors and Fine-Mapping the Snn3-B1 Locus Conferring Sensitivity to the Effector SnTox3
Source: Front Plant Sci. 2018 Jul 4;9:881. doi: 10.3389/fpls.2018.00881 (PMC6039772; doi:10.3389/fpls.2018.00881)
Supplement: Supplementary file 2 [file Table_2.PDF]

Supplementary Table 2. Highly significant (*P*<0.01, Bonferroni corrected, $-\log_{10}P=6.41$) SNPs identified in the AM panel for, (A) SnToxA sensitivity, (B) SnTox1. †IWGSC = IWGSC RefSeq v1.0. Chr = chromosome. U = not on the genetic map (Gardner *et al.* 2016). ‡Second best group homoeologue BLASTn hit. £Third best homoeologue BLASTn hit. Consensus chromosome: genetic map chromosome allocation supersedes physical map chromosome allocation. $ Chromosome allocation previously determined using the SNP as a trait, and localising to a chromosome by trait mapping (Gardner *et al.* 2016).

**Marker**	**Chr**	**Pos (cM)**	**$-\log_{10}P$**	**IWGSC† chr, bp**	**IWGSC† gene model**
**A**					
Tdurum_contig25513_195	5B	134.914	31.51127	5B, 546827158‡	TraesCS5B01G368500
tplb0027f13_1346	5B	135.9196	31.51127	5B,546827934	TraesCS5B01G368500
tplb0027f13_1493	5B	137.93	31.51127	5B, 546827787	TraesCS5B01G368500
Excalibur_rep_c105815_305$	U	U	31.51127	5B, 546849099	TraesCS5B01G368600
BobWhite_c48435_165	5B	134.9146	31.51127	5B, 565753994	TraesCS5B01G368500
Tdurum_contig12066_126	5B	136.9246	31.51127	5B, 546826482	TraesCS5B01G368500
Tdurum_contig12066_247	5B	136.9246	31.51127	5B, 546826603	TraesCS5B01G368500
wsnp_Ku_c40334_48581010	5B	134.9146	31.34971	5B, 546849099	TraesCS5B01G368600
BS00010590_51	5B	134.9146	29.45209	5B, 546704021‡	TraesCS5B01G368300
IACX9261	5B	133.9095	29.45209	5B, 546704036	TraesCS5B01G368300
wsnp_Ku_c3102_5810751	5B	132.402	10.72879	5B, 545399206	TraesCS5B01G366500
wsnp_Ku_c3102_5811860	5B	132.4020	10.72879	5B, 545403388	TraesCS5B01G366500
Kukri_c54078_114	5B	132.9046	10.41144	5B, 545406716	TraesCS5B01G366700
Ex_c13277_2025	5B	133.4071	10.26886	5B, 545801009	TraesCS5B01G367100
Kukri_c17396_2448	5B	138.4322	10.26886	5B, 545800013	TraesCS5B01G367100
Excalibur_c37642_1416	5B	137.4272	10.26886	5B, 545800481£	TraesCS5B01G367100
Kukri_c90424_72	5B	135.4171	10.26886	5B, 545577148	TraesCS5B01G366900
Ra_c38583_333	5B	136.4221	10.26886	5B, 545798143‡	TraesCS5B01G367100
Kukri_rep_c113115_424	5B	134.4121	10.26886	5B, 545799768	TraesCS5B01G367100
Kukri_rep_c113115_261	5B	137.4272	10.26886	5B, 545800031	TraesCS5B01G367100
Excalibur_c6649_769	5B	131.3919	9.95215	5B, 543613398	TraesCS5B01G364900
RAC875_c1565_1098	5B	131.3919	9.95215	5B, 543620696	TraesCS5B01G364900
wsnp_Ex_c8659_14515623	5B	132.402	9.51562	5B, 545403204	TraesCS5B01G366500
Excalibur_c6649_304	5B	131.3919	8.955087	5B, 543612787	TraesCS5B01G364900

Excalibur_c6649_794	5B	128.3459	8.955087	5B, 543613423	TraesCS5B01G364900
IACX7649	5B	123.06	7.557502	5B, 539294892‡	TraesCS5B01G359500
Kukri_c52_184$	U	U	7.557502	5B, 539293215	TraesCS5B01G359500
Kukri_c52_298	5B	123.0599	7.557502	5B, 539293329	TraesCS5B01G359500
Kukri_c52_1639	5B	123.0599	7.557502	5B, 539296455	TraesCS5B01G359500
Kukri_c29267_215	5B	125.0700	7.557502	5B, 539460175	TraesCS5B01G359700
**B**					
BS00093078_51	1B	2.5125	9.983884	1B, 1204018	TraesCS1B01G000800
BS00022296_51	1B	10.4169	8.158523	1B, 464668£	TraesCS1B01G000200
BS00030768_51	1B	10.9194	7.920517	1B, 5855088	TraesCS1B01G012000
Kukri_c37738_417	1B	10.4169	7.917393	1B, 5519895‡	None
Excalibur_c21898_1423	1B	1.5075	7.274817	1B, 1421208	TraesCS1B01G001600
BS00022020_51	1B	1.5075	7.218667	1B, 1421338	TraesCS1B01G001600
BS00064465_51	1B	9.9143	6.71616	1B, 2332679‡	None
