# Supplementary material for: Assessing European Wheat Sensitivities to Parastagonospora nodorum Necrotrophic Effectors and Fine-Mapping the Snn3-B1 Locus Conferring Sensitivity to the Effector SnTox3
Source: Front Plant Sci. 2018 Jul 4;9:881. doi: 10.3389/fpls.2018.00881 (PMC6039772; doi:10.3389/fpls.2018.00881)
Supplement: Supplementary file 3 [file Table_3.PDF]

Supplementary Table 3. Significant (Bonferroni corrected P=0.01, -$\log_{10}P$=6.41) markers for SnTox3 sensitivity identified in the AM panel by GWAS, using SNP GENE-3324_338 as a cofactor in the analysis. Physical map positions determined by BLASTn. TGACv1 gene models prefixed with TRIAE_CS42_5BS_TGACv1_. †SNP previously been shown to be located on chromosome 5B by using the genetically unmapped SNP as a trait, and localising to a chromosome by trait mapping (Gardner *et al.* 2016). Chr = chromosome.

**Marker name**	**-$\log_{10}P$**	**IWGSC RefSeq v1.0 chr, position (bp)**	**IWGSC RefSeq v1.0 gene model**	**TGACv1 chr, position (bp)**	**TGACv1 gene model**
**A**					
Excalibur_c13833_570	15.88	5A, 2897270†	TraesC5A01G004200	5A, 3748	729465_AA2172150
BS00066144_51	14.31	U, 318919829†	TraesCSU01G215500	5B, 2228	none
wsnp_Ex_c9301_15450818	13.43	U, 65429392†	TraesCSU01G073200	5B, 24489	424411_AA1389470
IACX7443	13.43	U, 96543638†	TraesCSU01G073300	5B, 17484	424411_AA1389460
Ra_c68425_1406	13.11	U, 65431410†	TraesCSU01G073200	5B, 22472	424411_AA1389470
Ra_c68425_1419	13.11	U, 65431423†	TraesCSU01G073200	5B, 22497	424411_AA1389470
RAC875_c7582_680	6.960	5D, 72270†	none	5D, 72270	456971_AA1480450
Ku_c10387_272	6.960	5B, 232228†	TraesCS5B01G00060	5B, 9061	424586_AA1390720
BS00032003_51	6.327	5B, 2559430†	TraesCS5B01G00160	5B, 122519	423227_AA1371660
