# Supplementary material for: Assessing European Wheat Sensitivities to Parastagonospora nodorum Necrotrophic Effectors and Fine-Mapping the Snn3-B1 Locus Conferring Sensitivity to the Effector SnTox3
Source: Front Plant Sci. 2018 Jul 4;9:881. doi: 10.3389/fpls.2018.00881 (PMC6039772; doi:10.3389/fpls.2018.00881)
Supplement: Supplementary file 4 [file Table_4.PDF]

Supplementary Table 4. Significant (*P*=0.05) markers for SnTox3 sensitivity identified by single marker analysis (SMA) in the MAGIC population. Chr = chromosome. U = not genetically mapped. Genetic map = Gardner *et al.* (2016). *P*-and *q*-values >2.2^{-16} are recorded in the analysis software as = 0. Therefore, SNPs with *P*- and *q-values* of zero are listed here in order of effect. ‡Chromosome allocations in brackets indicate SNPs that have previously been located on chromosome 5B via use of the SNP as a trait, and localising to a chromosome by trait mapping (Gardner *et al.* 2016). †While BLASTn for these SNPs identifies the best hit to be a homoeologues on chromosomes 5A or 5D, here the 5B hit is listed as the SNP has been allocated to chromosome 5B by Gardner *et al.* (2016).

**SNP**	***P*-value**	***q*-value**	**Effect**	**Chr‡, cM**	**IWGSC RefSeq v1.0 chr, bp**	**IWGSC RefSeq v1.0 Gene model**	**IWGSC RefSeq v1.0 annotation**
Excalibur_c47452_183	0	0	1.254	(5B), U	5B, 6654166	TraesCS5B01G005100	Ubiquitin-conjugating enzyme E2
GENE-3324_338	0	0	1.254	(5B), U	5B, 6647920†	TraesCS5B01G005000	P-loop containing nucleoside triphosphate hydrolases superfamily
BobWhite_c4838_58	0	0	1.249	(5B), U	5B, 6654053	TraesCS5B01G005100	Ubiquitin-conjugating enzyme E2
BS00091518_51	0	0	-1.241	(5B), U	5B, 6648547	TraesCS5B01G005000	P-loop containing nucleoside triphosphate hydrolases superfamily
BS00091519_51	0	0	-1.239	(5B), U	5B, 6648567	TraesCS5B01G005000	P-loop containing nucleoside triphosphate hydrolases superfamily
RAC875_c39204_91	0	0	0.951	(5B), U	5B, 6852650	none	U
BS00064297_51a	0	0	-0.939	(5B), U	5B, 6974807	TraesCS5B01G005600	transmembrane protein, putative (DUF594)
BS00064298_51a	0	0	-0.934	(5B), U	5B, 6974825	TraesCS5B01G005600	transmembrane protein, putative (DUF594)
Ex_c1846_1818a	0	0	-0.930	(5B), U	5B, 64736555	TraesCS5B01G059000	Protein kinase family protein
BS00067985_51	0	0	0.811	(5B), U	5A, 6799078	TraesCS5A01G010500	Elongation of fatty acids protein 3-like
Excalibur_c32189_998	0	0	-0.802	(5B), U	5B, 8344134	TraesCS5B01G007100	Cytochrome P450
BobWhite_c23714_130	0	0	-0.802	(5B), U	5B, 8918076†	TraesCS5B01G007800	SacI-like domain protein/WW domain protein
Kukri_rep_c70770_1937	0	0	0.802	(5B), U	5B, 8910549†	TraesCS5B01G007800	SacI-like domain protein/WW domain protein
BS00060650_51	0	0	0.801	(5B), U	5B, 8166684	none	U
BobWhite_rep_c63710_181	0	0	0.801	(5B), U	5B, 7541567	TraesCS5B01G006400	BEL1-like homeodomain protein 6

tplb0060p09_735	0	0	0.799	(5B), U	5B, 8343408	TraesCS5B01G007100	Cytochrome P450
Excalibur_c32189_941	0	0	0.798	(5B), U	5B, 8344077	TraesCS5B01G007100	Cytochrome P450
BS00079166_51	0	0	0.798	(5B), U	5B, 7706212	TraesCS5B01G006500	Lipoxygenase
wsnp_CAP11_rep_c4382_2067038	0	0	0.798	(5B), U	5B, 7706212	TraesCS5B01G006500	Lipoxygenase
BS00102828_51	0	0	0.797	(5B), U	5B, 8353214	TraesCS5B01G007200	Cytochrome P450
BS00024992_51	0	0	0.797	(5B), U	5B, 7937876	TraesCS5B01G006600	Serine protease HtrA-like
Excalibur_c6967_1852	0	0	-0.795	(5B), U	5B, 8916847	TraesCS5B01G007800	SacI-like domain protein/WW domain protein
BS00024993_51	0	0	0.793	(5B), U	5B, 7937753	TraesCS5B01G006600	Serine protease HtrA-like
BS00010153_51	0	0	0.791	(5B), U	5B, 8905311	TraesCS5B01G007800	SacI-like domain protein/WW domain protein
Excalibur_c6967_553	0	0	0.791	(5B), U	5B, 8910639	TraesCS5B01G007800	SacI-like domain protein/WW domain protein
Excalibur_c6967_1233	0	0	-0.791	(5B), U	5B, 8911807	TraesCS5B01G007800	SacI-like domain protein/WW domain protein
RFL_Contig4979_965	0	0	-0.791	(5B), U	5B, 8918212	TraesCS5B01G007800	SacI-like domain protein/WW domain protein
Kukri_c65921_274	0	0	-0.791	(5B), U	5B, 8917489	TraesCS5B01G007800	SacI-like domain protein/WW domain protein
BS00062280_51	0	0	-0.717	(5B), U	5B, 10060262	TraesCS5B01G010200	Maturase K
wsnp_Ex_c2459_4591695	0	0	0.678	(5B), U	5B, 12324424	TraesCS5B01G012300	Enolase
BS00009810_51	0	0	0.636	(5B), U	5B, 10265142	TraesCS5B01G010400	chitin synthase, putative (DUF1218)
BS00067550_51	0	0	-0.632	(5B), U	5B, 11043290	TraesCS5B01G011000	Coenzyme PQQ synthesis protein F
wsnp_Ex_c831_1625061	0	0	-0.632	(5B), U	5B, 10524933	TraesCS5B01G010900	fiber (DUF1218)
BS00022525_51	0	0	0.632	(5B), U	5B, 10444933	TraesCS5B01G010800	ERD (Early-responsive to dehydration stress) family protein
BS00083715_51	0	0	0.632	(5B), U	5B, 10445103	TraesCS5B01G010800	ERD (Early-responsive to dehydration stress) family protein
Tdurum_contig14130_245	0	0	0.632	(5B), U	5B, 10444793	TraesCS5B01G010800	ERD (Early-responsive to dehydration stress) family protein
Tdurum_contig14130_3	0	0	0.632	(5B), U	5B, 10444723	TraesCS5B01G010800	ERD (Early-responsive to

15							dehydration stress) family protein
wsnp_Ex_c607_1204908	0	0	0.632	(5B), U	5B, 10438196	FGENESH_183291_188075_Gene_0023	U
RFL_Contig4911_357	0	0	0.630	(5B), U	5B, 10449146	TraesCS5B01G010800	ERD (Early-responsive to dehydration stress) family protein
wsnp_Ex_c607_1204733	0	0	-0.630	(5B), U	5B, 10438371	TraesCS5B01G010700	ERD (Early-responsive to dehydration stress) family protein
BS00107529_51	4.44^{-16}	2.24^{-13}	-0.610	(5B), U	5B, 10169315	TraesCS5B01G010300	1,3-beta-glucan synthase component (DUF1218)
RAC875_c7582_680	2.89^{-15}	1.42^{-12}	0.577	(5B), U	5B, 2058821	TraesCS5B01G002000LC	U
BS00065164_51	7.66^{-15}	3.68^{-12}	0.571	(5B), U	5B, 4862088	none	U
Ku_c10387_272	1.19^{-14}	5.58^{-12}	-0.570	(5B), U	5B, 232228	TraesCS5B01G000600	Microtubule-associated protein 70-2
Excalibur_c74858_243	7.12^{-09}	3.27^{-06}	0.391	(5B), U	5B, 13190710	TraesCS5B01G013300	Pyridine nucleotide-disulfide oxidoreductase domain-containing protein 2
wsnp_Ex_c12927_20480163	2.58^{-07}	11.3306^{-5}	0.349	5B, 2.276	5B, 16423462	TraesCS5B01G017800	Anaphase-promoting complex subunit 5
wsnp_Ku_c5308_9450093	2.57^{-07}	11.3306^{-5}	0.346	5B, 2.276	5B, 16422084	TraesCS5B01G017800	Anaphase-promoting complex subunit 5
GENE_3277_145	3.00^{-07}	12.9006^{-5}	0.346	5B, 2.276	5B, 16423444[†]	TraesCS5B01G017800	Anaphase-promoting complex subunit 5
wsnp_Ex_rep_c110196_92676847	3.48^{-07}	14.6593^{-5}	0.345	5B, 2.276	5B, 16422835	TraesCS5B01G017800	Anaphase-promoting complex subunit 5
BS00079185_51	4.37^{-07}	18.0402^{-5}	0.498	(5B), U	5B, 15553205	TraesCS5B01G016900	Nucleolar complex protein 2-like
IAAV731	5.30^{-07}	20.2588^{-5}	0.489	(5B), U	5B, 13725863	TraesCS5B01G014500	Haloacid dehalogenase-like hydrolase domain-containing
Tdurum_contig53796_360	5.30^{-07}	20.2588^{-5}	0.489	(5B), U	5B, 13724854	TraesCS5B01G014500	Haloacid dehalogenase-like hydrolase domain-containing
Tdurum_contig8695_379	5.30^{-07}	20.2588^{-5}	0.489	(5B), U	5B, 13725887	TraesCS5B01G014500	Haloacid dehalogenase-like hydrolase domain-containing protein
RAC875_c47212_309	5.30^{-07}	20.2588^{-5}	-0.489	(5B), U	5B, 13726107	TraesCS5B01G014500	Haloacid dehalogenase-like hydrolase domain-containing

							protein
BS00022336_51	7.24^{-07}	26.6859^{-5}	0.484	5B, 3.2862	5B, 15553300	TraesCS5B01G016900	Nucleolar complex protein 2-like
RAC875_c39430_181	7.16^{-07}	26.6859^{-5}	0.479	(5B), U	5B, 87538	TraesCS5B01G000300	NADH dehydrogenase
BS00025784_51	7.76^{-07}	28.1007^{-5}	-0.485	5B, 3.2862	5B, 15553300	TraesCS5B01G016900	Nucleolar complex protein 2-like
BS00080746_51	8.39^{-07}	28.3898^{-5}	0.481	5B, 3.2862	5B, 14509045	TraesCS5B01G017900LC	U
BS00064044_51	8.39^{-07}	28.3898^{-5}	-0.481	5B, 3.2862	5B, 14718395	none	U
JD_c51907_59	8.39^{-07}	28.3898^{-5}	-0.481	5B, 3.2862	5B, 14510777	TraesCS5B01G017900LC	U
Ex_c37410_1026	8.39^{-07}	28.3898^{-5}	-0.481	(5B), U	5B, 14510777	TraesCS5B01G017900LC	U
wsnp_Ex_c9301_15450818	1.05^{-06}	34.9565^{-5}	-0.466	(5B), U	U, 65429353	TraesCSU01G073200	Plasma membrane ATPase
RAC875_c43191_473	1.08^{-06}	35.3846^{-5}	-0.486	(5B), U	6D, 68333368	TraesCS6D01G104700	Splicing factor 3B subunit 1
RAC875_rep_c114631_389	1.65^{-06}	53.2151^{-5}	0.475	5B, 3.7887	5B, 16034641	TraesCS5B01G017300	Splicing factor 3B subunit 1
BS00034658_51	1.73^{-06}	54.9368^{-5}	0.460	(5B), U	5B, 21006	TraesCS5B01G000100	Sucrose transporter protein
BS00022107_51	1.77^{-06}	55.3554^{-5}	-0.321	5B, 15.6783	5B, 34719414	TraesCS5B01G031600	Disease resistance protein RPM1
BS00065732_51	2.06^{-06}	59.6296^{-5}	0.471	5B, 3.7887	5B, 16033751	TraesCS5B01G017300	Splicing factor 3B subunit 1
Kukri_c46276_63	2.06^{-06}	59.6296^{-5}	0.471	5B, 3.7887	5B, 16002417	TraesCS5B01G017100	PI-PLC X domain-containing protein
BS00066144_51	2.08^{-06}	59.6296^{-5}	-0.454	(5B), U	5B, 6023362	TraesCS5B01G004100	BED zinc finger,hAT family dimerization domain, putative
IACX7443	2.08^{-06}	59.6296^{-5}	-0.454	(5B), U	U, 65436455	TraesCSU01G073300	Oxidoreductase, putative
Ra_c68425_1419	2.08^{-06}	59.6296^{-5}	-0.454	(5B), U	U, 65431423	TraesCSU01G073200	Plasma membrane ATPase
Ra_c68425_1406	2.08^{-06}	59.6296^{-5}	0.454	(5B), U	U, 65431410	TraesCSU01G073200	Plasma membrane ATPase
BS00022602_51	2.16^{-06}	61.0747^{-5}	-0.319	5B, 14.6733	5B, 34253053	TraesCS5B01G031200	Dihydrolipoamide acetyltransferase component of pyruvate dehydrogenase complex
BS00088218_51	2.49^{-06}	69.4542^{-5}	-0.461	(5B), U	5B, 13428619	TraesCS5B01G013900	MscS Mechanosensitive ion channel
BobWhite_c4852_323	4.94^{-06}	135.9554^{-5}	-0.304	5B, 27.7951	5B, 68359041	TraesCS5B01G061000	Glycosyltransferase
BS00064042_51	5.03^{-06}	136.6108^{-5}	0.421	(5B), U	5B, 14718207	none	U
Excalibur_rep_c104354	8.25^{-06}	221.1536^{-5}	-0.432	5B, 3.7887	5B, 16035052[†]	TraesCS5B01G017300	Splicing factor 3B subunit 1

_205							
Excalibur_c13833_570	8.63^{-06}	228.3741^{-5}	-0.413	(5B), U	5A, 2897270	TraesCS5A01G004200	Plasma membrane ATPase
BS00032003_51	9.12^{-06}	238.286^{-5}	-0.421	(5B), U	5B, 2559430	TraesCS5B01G001600	Ethylene receptor
wsnp_Ku_c2119_4098330	1.20^{-05}	307.0978^{-5}	-0.358	6B, 14.4725	NA	NA	NA
Kukri_c60322_490	1.22^{-05}	0.00307097	0.299	5B, 6.8824	5B, 19438660	TraesCS5B01G019600	NA
wsnp_Ex_c35103_43312537	1.22^{-05}	0.00307097	0.299	5B, 6.8824	NA	NA	NA
wsnp_Ex_c1143_2195442	1.45^{-05}	0.00358760	-0.355	6B, 12.7118	NA	5B, 19438660	TraesCS5B01G019600
wsnp_Ex_c1143_2195598	1.46^{-05}	0.00358760	-0.353	6B, 12.2093	NA	NA	NA
Ex_c68034_498	1.48^{-05}	0.00359396	-0.446	(5B), U	5B, 21535004	TraesCS5B01G023300	Cationic amino acid transporter, putative
wsnp_BE499835B_Ta_2_5	2.24^{-05}	0.00537626	-0.433	5B, 12.1504	5B, 20832543	TraesCS5B01G022300	NA
CAP8_rep_c5825_165	2.45^{-05}	0.00581269	0.389	5B, 3.2862	5B, 15054913	TraesCS5B01G015700	Thaumatin-like protein
BobWhite_c26680_62	2.50^{-05}	0.00586392	-0.441	(5B), U	5B, 13179540	TraesCS5B01G013200	RNA-binding protein
BS00062617_51	4.63^{-05}	0.01073795	-0.415	5B, 12.1504	5B, 21538528	TraesCS5B01G023300	NA
RAC875_c29907_293	5.10^{-05}	0.01169656	-0.275	(5B), U	5B, 15321551	TraesCS5B01G016700	NA
BobWhite_c5887_1277	5.61^{-05}	0.01272483	-0.413	5B, 14.1707	5B, 21536610	TraesCS5B01G023300	NA
Excalibur_c4468_654	7.12^{-05}	0.01546988	-0.408	5B, 14.17074	5B, 21750048	TraesCS5B01G023700	NA
Excalibur_rep_c69526_921	7.12^{-05}	0.01546988	-0.408	5B, 14.17074	5B, 21750530[†]	TraesCS5B01G023700	NA
Excalibur_c3730_546	7.12^{-05}	0.01546988	0.408	5B, 14.17074	5B, 217514057	TraesCS5B01G023700	NA
Tdurum_contig48100_260	7.01^{-05}	0.01546988	-0.401	5B, 14.17074	5B, 21749572	TraesCS5B01G023700	NA
BS00067701_51	7.60^{-05}	0.01634079	0.393	5B, 15.1757	5B, 19458998	none	U
RAC875_c29907_115	7.88^{-05}	0.01676554	-0.271	(5B), U	5B, 15321856	TraesCS5B01G016700	NA
BS00068528_51	7.96^{-05}	0.01676554	-0.255	5B, 12.1504	5B, 23331598	TraesCS5B01G032900LC	NA
Excalibur_c29975_333	8.98^{-05}	0.01872284	0.269	(5B), U	5B, 15322178	TraesCS5B01G016700	NA
Kukri_c9898_1766	11.459	0.02365293	-0.393	2B, 356.6633	U, 31518378	TraesCSU01G037700	NA

	$^{-5}$						
BS00093771_51	13.009^{-5}	0.02658560	0.273	(5B), U	5B, 25666832	AUGUSTUS_wheat_440821_446095_gene_007	NA
Excalibur_c3730_2098	17.911^{-5}	0.03521059	-0.375	5B, 14.1707	NA	NA	NA
Kukri_c10970_573	17.911^{-5}	0.03521059	-0.375	5B, 14.1707	NA	NA	NA
RAC875_c6565_129	17.911^{-5}	0.03521059	-0.375	5B, 14.1707	NA	NA	NA
IAAV7134	17.911^{-5}	0.03521059	0.375	5B, 14.1707	5B, 21750006[†]	TraesCS5B01G023700	NA
BS00036421_51	19.136^{-5}	0.03726323	0.233	4D, 40.6091	NA	NA	NA
BS00022127_51	20.381^{-5}	0.03824327	-0.282	7B, 9.4272	NA	NA	NA
Ex_c24068_652	20.381^{-5}	0.03824327	-0.282	7B, 9.4272	NA	NA	NA
Excalibur_c8486_419	20.381^{-5}	0.03824327	-0.282	7B, 9.4272	NA	NA	NA
Excalibur_c8486_471	20.381^{-5}	0.03824327	-0.282	7B, 9.4272	NA	NA	NA
RAC875_rep_c111261_94	22.82^{-5}	0.04243510	0.371	5B, 15.1757	NA	NA	NA
Excalibur_c15671_87	24.735^{-5}	0.04558601	0.374	2B ,57.1658	NA	NA	NA
BS00010491_51	25.454^{-5}	0.04649485	-0.366	5B, 14.1707	NA	NA	NA
IAAV8607	27.009^{-5}	0.04890215	0.239	5B, 30.8204	5B, 55495326	TraesCS5B01G077600LC	U
