# Supplementary material for: Assessing European Wheat Sensitivities to Parastagonospora nodorum Necrotrophic Effectors and Fine-Mapping the Snn3-B1 Locus Conferring Sensitivity to the Effector SnTox3
Source: Front Plant Sci. 2018 Jul 4;9:881. doi: 10.3389/fpls.2018.00881 (PMC6039772; doi:10.3389/fpls.2018.00881)
Supplement: Supplementary file 5 [file Table_5.PDF]

Supplementary Table 5. Comparison of allele call for marker Excalibur_c47452_183 generated using the 90k array and the KASP marker developed in this study. U = missing data.

Association mapping panel accession	90k array genotype call	KASP genotype call
S1591_AC_BARRIE	A:A	A:A
S185_SICCO	A:A	A:A
S249_AINTREE	G:G	G:G
S337_SANDOWN	A:A	A:A
S494_SOLITAIRE	A:A	A:A
S497_JERICO	G:G	G:G
S53_MARIS_BUTLER	G:G	G:G
S547_WEMBLEY	A:A	A:A
U100273_CAPELLE_DESPREZ	A:A	A:A
U100347_COURTOT	A:A	A:A
U100546_GARNET	G:G	G:G
U101082_SELKIRK	A:A	A:A
U101237_VIKING	A:A	A:A
U112030_TAMBOR	A:A	A:A
U112495_ISENGRAIN	G:G	G:G
U112544_AMAROK	A:A	A:A
U112547_ARONDE	A:A	A:A
U112943_LORRAINE	A:A	A:A
U9000_80_30_VERSAILLES	A:A	A:A
U9004_CARSTENS_VIII	G:G	G:G
U9008_ELYSEE	A:A	A:A
U9013_HYBRID_46	A:A	A:A
U9016_KOGA_1	A:A	A:A
U9020_MARNE_DESPREZ	A:A	A:A
U9028_OBELISK	G:G	G:G
U9032_RIEBESEL_57/41	G:G	G:G
U9033_STELLA	A:A	A:A
U9036_THATCHER	A:A	A:A
W1020_TILBURI	A:A	A:A
W1031_FALSTAFF	A:A	A:A
W105_MARIS_MARKSMAN	A:A	A:A
W1088_ROSETTE	A:A	A:A
W109_MEGA	A:A	A:A
W1092_SHAMROCK	G:G	G:G
W1108_DATUM	A:A	A:A
W1115_DICKINS	A:A	A:A
W1206_GOLDLACE	A:A	A:A
W1233_ARK	A:A	A:A
W1267_ANGLO	A:A	A:A
W1277_FRELON	U	G:G
W1286_STORM	A:A	A:A
W1310_CAPNOR	A:A	A:A
W1321_PR21R60	A:A	A:A
W1326_TELLUS	G:G	G:G
W1328_WIZARD***	A:A	A:A
W1407_AWARD	A:A	A:A
W1435_SW_TATAROS	U	G:G
W1438_CAPHORN	A:A	A:A
W1439_DART*	A:A	A:A

W1441_MONUMENT	A:A	A:A
W1545_ZEBEDEE	A:A	A:A
W1546_GATSBY	G:G	G:G
W1549_FASTNET	A:A	A:A
W1550_DOVER	A:A	A:A
W1625_BENEDICT	A:A	A:A
W1668_MAXWELL	A:A	A:A
W169_ALCEDO	A:A	A:A
W1695_BOWINDO	A:A	A:A
W173_FLANDERS	A:A	A:A
W1737_JB_DIEGO	A:A	A:A
W1760_GALTIC	G:G	G:G
W1806_TIMARU	A:A	A:A
W1830_ROCHFORT	A:A	A:A
W1858_SANTANA	G:G	G:G
W1909_KWS_GYMNAST	A:A	A:A
W192_DURIN	A:A	A:A
W205_KADOR	A:A	A:A
W23_TOMMY	A:A	A:A
W243_AQUILA	A:A	A:A
W260_ANVIL	A:A	A:A
W265_COPAIN	A:A	A:A
W271_IONA	A:A	A:A
W272_SENTRY	A:A	A:A
W286_HERALD	A:A	A:A
W289_SHIRE	A:A	A:A
W296_GRANTA	A:A	A:A
W328_DISPONENT	G:G	G:G
W371_JENA	G:G	G:G
W4_BOUQUET	A:A	A:A
W509_BOXER	A:A	A:A
W584_SARSEN	A:A	A:A
W63_BENNO	G:G	G:G
W65_MARIS_PLOUGHMAN	A:A	A:A
W67_MARIS_TEMPLAR	G:G	G:G
W671_PASTICHE	A:A	A:A
W682_URBAN	G:G	G:G
W724_EKLA	A:A	A:A
W732_DEAN	G:G	G:G
W785_ARISTOCRAT	A:A	A:A
W803_FLETUM	G:G	G:G
W811_FENDA	G:G	G:G
W834_SOISSONS	G:G	G:G
W900_TRAWLER	A:A	A:A
W938_SHANNON	G:G	G:G
W966_RUBENS	A:A	A:A
