# Supplementary material for: Assessing European Wheat Sensitivities to Parastagonospora nodorum Necrotrophic Effectors and Fine-Mapping the Snn3-B1 Locus Conferring Sensitivity to the Effector SnTox3
Source: Front Plant Sci. 2018 Jul 4;9:881. doi: 10.3389/fpls.2018.00881 (PMC6039772; doi:10.3389/fpls.2018.00881)
Supplement: Supplementary file 6 [file Table_6.PDF]

Supplementary Table 6. Gene content within the 320 kb region on chromosome 5B containing the most significant ($-\log_{10}P>16$, effect >1) SNPs associated with the *Snn3-B1* locus identified by GWAS in the AM panel and SMA in the MAGIC population. Physical map coordinates, gene model IDs and gene annotations: IWGSC RefSeq v1.0. All gene models are represented by their first transcript form. †Low confidence IWGSC RefSeq v1.0 gene models. ‡Identified in the AM panel by GWAS or in the MAGIC population by SMA.

Gene ID	CDS start (bp)	CDS stop (bp)	Strand	CDS (bp)	Protein (aa)	Significant SNPs‡	Gene annotation
TraesCS5B01G005000	6645300	6648610	+	2067	689	GENE-3324_338<br>BS00091518_51<br>BS00091519_51	P-loop containing nucleoside triphosphate hydrolases superfamily protein
TraesCS5B01G005100	6650171	6654128	+	486	162	Excalibur_c47452_183<br>BobWhite_c4838_58	Ubiquitin-conjugating enzyme E2
TraesCS5B01G005200†	6659930	6677348		1224	408		Serine protease HtrA-like
TraesCS5B01G005300	6834483	6837264	-	726	242		Expressed protein, 5'-partial
TraesCS5B01G005400†	6918180	6927740	-	2019	673		Receptor protein kinase-like protein
TraesCS5B01G005500†	6968208	6970169	+	1962	654		transmembrane protein, putative (DUF594)
TraesCS5B01G005600†	6973387	6975396	+	2010	670	BS00064297_51b<br>BS00064298_51b	transmembrane protein, putative (DUF594)
