# Supplementary material for: Assessing European Wheat Sensitivities to Parastagonospora nodorum Necrotrophic Effectors and Fine-Mapping the Snn3-B1 Locus Conferring Sensitivity to the Effector SnTox3
Source: Front Plant Sci. 2018 Jul 4;9:881. doi: 10.3389/fpls.2018.00881 (PMC6039772; doi:10.3389/fpls.2018.00881)
Supplement: Supplementary file 7 [file Table_7.PDF]

Supplementary Table 7. Comparison of minor SnTox3 sensitivity QTL identified by CIM in the MAGIC population with to previously identified QTL on relevant chromosomes (chr). U = no query sequence available. [1]Phan *et al.* 2016, seedling inoculation with *P. nodorum* isolate SN15. [2]Phan *et al.* 2016, seedling inoculation with isolate *tox1-6*. [3]Phan *et al.* (2016), seedling inoculation with isolate *toxa13*. [4]Phan *et al.* (2016), infiltration with culture filtrate from *toxa13*. [5]Ruud *et al.* (2017b), field SNB resistance. [†]Published SNB resistance, effector sensitivity or isolate culture filtrate sensitivity QTL.

SnTox3 QTL	Chr	SNPs flanking QTL peak	IWGSC position (Mb)	QTL[†] on relevant chromosomes: QTL name, flanking markers	IWGSC positions (Mb) of flanking markers
QTox3.niab-2A.1	2A	BS00070979_51	758.692	*Qsnb.cur–2AS.1*, gwm515 to gwm328[1]	172.678 to 584.228
		Excalibur_c20478_641	758.554	*Qsnb.cur–2AS.1*, gwm339 to gwm312[2]	112.746 to 709.048
				Qsnb.cur–2AS.1, tPt-8937 to gwm312[3]	U to 709.048
				Qsnb.cur–2AS.1, wPt-9320 to gwm328[4]	U to 584.228
QTox3.niab-3B.1	3B	wsnp_Ex_c11246_18191331	67.942	3B1.2, wsnp_BE445348B_Ta_2_1[5]	57.483
		wsnp_Ex_c22401_31592784	68.466		
QTox3.niab-6A.1	6A	BobWhite_c13839_135	23.833		
		IACX7801	22.524		
