# Supplementary material for: Assessing European Wheat Sensitivities to Parastagonospora nodorum Necrotrophic Effectors and Fine-Mapping the Snn3-B1 Locus Conferring Sensitivity to the Effector SnTox3
Source: Front Plant Sci. 2018 Jul 4;9:881. doi: 10.3389/fpls.2018.00881 (PMC6039772; doi:10.3389/fpls.2018.00881)

Supplementary Figure 1. Histogram of SnTox3 sensitivity in the NIAB Elite MAGIC population (number of progeny = 643, SnTox3 score of each progeny line is the mean of 3-4 reps).

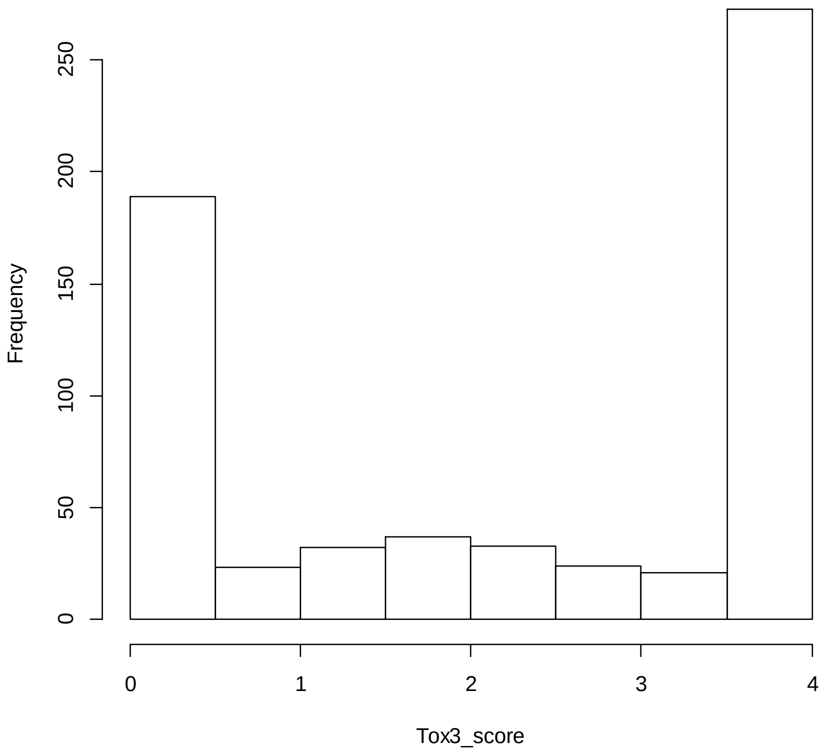

Supplement: Supplementary file 8 [file Image_1.PDF]
